# Crystalline and amorphous structure selectivity of ignoble high-entropy alloy nanoparticles during laser ablation in organic liquids is set by pulse duration

**DOI:** 10.3762/bjnano.16.84

**Published:** 2025-07-17

**Authors:** Robert Stuckert, Felix Pohl, Oleg Prymak, Ulrich Schürmann, Christoph Rehbock, Lorenz Kienle, Stephan Barcikowski

**Affiliations:** 1 Technical Chemistry I and Center for Nanointegration Duisburg-Essen (CENIDE), University of Duisburg-Essen, Universitaetsstr. 7, 45141 Essen, Germany; 2 Institute for Material Science, Synthesis and Real Structure, Faculty of Engineering, Christian-Albrechts University of Kiel, Kaiserstraße 2, 24143 Kiel, Germany; 3 Inorganic Chemistry and Center for Nanointegration Duisburg-Essen (CENIDE), University of Duisburg-Essen, Universitaetsstr. 7, 45141 Essen, Germany; 4 Kiel Nano, Surface and Interface Science (KiNSIS), Christian-Albrechts University of Kiel, Christian-Albrechts-Platz 4, 24118 Kiel, Germany

**Keywords:** amorphous, cantor alloy, compositionally complex alloy, complex solid solution, EELS, electron energy loss spectroscopy, laser processing in liquids, multicomponent alloy, STEM-EDX, selected area electron diffraction, X-ray diffraction

## Abstract

High-entropy alloy nanoparticles (HEA NPs) represent a promising material class with significant potential in various applications, such as heterogeneous catalysis or magnetic devices. This is due to their exceptional compositional tunability arising from the synergistic interplay of multiple elements within a single particle. While laser-synthesized, surfactant-free colloidal HEA NPs have already been reported, the underlying formation mechanism remains unknown, particularly the underexplored preference of amorphous over crystalline structures warrants further investigation. Herein, we present a systematic study of laser-generated equimolar CrMnFeCoNi nanoparticles, focusing on structural differences, arising from varying pulse durations during synthesis in organic solvents (acetone, ethanol, acetonitrile). In a systematic experimental series using high-resolution transmission electron microscopy, scanning transmission electron microscopy with energy-dispersive X-ray spectroscopy, selected-area electron diffraction, X-ray diffraction, electron energy loss spectroscopy, in situ heating, post-irradiation experiments, and differential scanning calorimetry we demonstrate that a pulse-duration-driven structural difference occurs during laser ablation in liquid is observable to the three utilized solvents. While picosecond-pulsed laser ablation in liquid produces polycrystalline HEA NPs, nanosecond-pulsed laser ablation favors a metastable amorphous structure. Particle cores in all cases exhibit a homogeneous distribution of the metals Cr, Mn, Fe, Co, and Ni, while particle shells were found to vary between manganese-enriched oxide layers and thin graphitic carbon coatings. The discovery of the structure-directing mechanism allows one to select between crystalline or amorphous HEA NP products, simply by choice of the laser pulse duration in the same, well-scalable setup, giving access to colloidal particles that can be further downstream processed to heterogeneous catalysts or magnets. In that context, the outstanding temperature stability up to 375 °C (differential scanning calorimetry) or 500 °C (transmission electron microscopy) may motivate future application-relevant work.

## Introduction

High-entropy alloys (HEAs), also referred to as compositionally complex solid solutions (CCSS) [1], are of great interest in various applications in magnetic technologies [2–3] and electrocatalysis [2,4], derived from the combination of single-element properties which results in enhanced features compared to single-element properties [5]. High-entropy alloy nanoparticles (HEA NPs) constitute a relatively new class of nanomaterials, usually consisting of single-phase solid solutions made of five or more elements, forming relatively simple face-centered cubic [6–8] (fcc) or body-centered cubic [9–10] (bcc) crystal structures, stabilized by the configurational part of Gibbs-free energy. It is worth noting that high-entropy stabilization is highly disputed and the often discussed core effects of HEA do not apply to every element system [11–12]. Still we decided to refer to the term HEA in this work as it is deeply rooted within the community, despite the existence of names such as CCSS, complex concentrated alloys, compositionally complex alloys, derived from the critical view of the high-entropy effect [1]. The symbiosis of multiple elements leading to potential highly effective properties was already shown in energy applications [13], particularly in the field of heterogeneous catalysis, boosting efficiencies in ammonia decomposition [4], oxygen evolution reactions (OER) [14], oxygen reduction reactions (ORR) [15], in CO oxidation [16], and in CO_2_ and CO reduction [17]. This high activity in catalysis has been attributed to adsorption energy distribution patterns (AEDP) during catalytic reactions [18], which may be tuned based on density functional theory calculations of binding energies and machine learning algorithms for an efficient catalyst design [15,17,19]. The synthesis of HEA NPs has been realized by many methods, including carbothermal shock synthesis (CTS) [20–21], chemical reduction [22–23], fast-moving bed pyrolysis [24], and solvothermal methods [25] as addressed in several review articles [26–29]. In brief, the usual structures obtained when synthesizing HEA NPs are mostly fcc and bcc. Applying CTS, Yao et al. reported quinary, senary, septenary, and even octonary HEA NPs, all forming an fcc lattice [20]. In addition, the synthesis of the CrMnFeCoNi system by arc-discharge plasma method also lead to the formation of fcc HEA NPs [7]. The addition of Al to the high-entropy alloy system was shown to stabilize a bcc-type structure in the synthesized HEA NPs [14,30], while amorphous FeCoNiCrMo*_x_* HEA NPs could also be synthesized by inert gas condensation as stated very recently by Zhou et al. [31]. However, none of these aforementioned synthesis techniques give access to colloidal HEA NPs but to structures bound to an often very specific support material or limited to gas phase conditions. An approach for the synthesis of colloidal HEA NPs is magnetron sputtering, though special requirements such as vacuum stable solvents (e.g., ionic liquids) limit its widespread use [32] and scalability is limited. Colloidal NP products have several advantages. For instance, in the fabrication of heterogeneous catalysts, colloids are more flexible as the support material is not predefined by the synthesis route but can be freely chosen. This may be highly relevant as previous studies report on the facile supporting of laser-generated NPs onto multiple carrier materials such as carbon [33–35], metal oxides [36–37], or metal electrodes [38–39], using electrostatic [37], diffusive [40], or electrophoretic [39] pathways. Additionally, in contrast to form-in-place-methods such as CTS [20], the NP size does not depend on the loading [40].

Nanoparticle generation by laser synthesis and processing of colloids (LSPC) [41–44] provides nanoparticles dispersed in liquids without the addition of common additives (e.g., citrate [45], tensides [46], polymers [47]) or support material to stabilize the particles with high variability on the used solvent, yielding colloidal nanoparticles with productivities up to 8 g/h [48]. The throughput linearly scales with laser power [49], so that energy-specific mass productivity values are useful scaling factors. Here, the experimentally determined values of 9.7 µg/J [48] align with those from computational works (5–7 µg/J) [48,50]. Depending on the set goal, colloidal nanoparticles can be synthesized and/or processed by laser ablation in liquid (LAL) [51–55], laser fragmentation in liquid (LFL) [56–57], and laser reduction in liquid (LRL) [58–60], making LSPC an efficient method for nanoparticle research but also for scale-up, as it has been shown to be less expensive than wet chemically produced NPs after a break-even-point of 550 mg/h. Thus, it makes the laser system to be of low cost in large-scale processes [61]. Furthermore, procedures for LAL are usually not bound to strict limitations when it comes to pressure and solvent, as for example in magnetron sputtering techniques. Colloidal HEA NPs by LSPC were first reported in 2019 by Waag et al. [35], where CrMnFeCoNi NPs were produced in ethanol, using a picosecond-pulsed laser for ablation (ps-LAL), yielding colloids of HEA NPs with a mean diameter below 10 nm and productivities of 3 g/h. Both the bulk target and the NPs showed a crystalline fcc phase with a lattice parameter of 3.58 Å and a solid solution structure. Löffler et al. investigated the very same element system enriched with Mn, again made by ps-LAL in ethanol. They reported particle mean diameters below 10 nm and a solid solution fcc structure, whereas this study was more focused on the applicability in catalysis [18]. By irradiating metal chloride salts, premixed in ethanol and then added to a hexane solution with oleic acid present, Wang et al. successfully synthesized PtIrCuNiCr NPs with a narrow size distribution and uniformly mixed elements within the generated nanoparticles [62]. Recently, Tahir et al. conducted ps-LAL on the CrMnFeCoNi system in ethanol and varied production methods for the bulk targets, showing uniformly mixed HEA NPs with an fcc structure and indications for a minor fraction of oxidized manganese [63]. Johny et al. used nanosecond-pulsed LAL in acetonitrile to fabricate colloidal CrMnFeCoNi and CrMnFeCoNiMo HEA NPs with an amorphous structure and only minor contributions from crystalline phases [34]. As LSPC methods are based on ultrashort (fs, ps) or short (ns) pulsed lasers, unparalleled high cooling rates occur [64–65], resulting in distinct NP undercooling [50], creating defect-rich NP structures [40,66–67]. Despite these high cooling rates, LSPC-fabricated metal or alloy NPs are usually crystalline. At the example of immiscible binary alloys, it has been clarified that LAL can be classified as a kinetically controlled synthesis method, with thermodynamic contributions at the nanoscale which set the final NP structure during cooling [68]. Hence, thermodynamically metastable NP structures are generated, which are stable at elevated temperatures [69] and even under harsh thermocatalytic [66] and electrocatalytic [67] reaction conditions.

Even though amorphous NPs are generally well studied [70–74], their synthesis by LSPC is still in its infancy as recently highlighted [75], though a few basic rules could be derived from previous LSPC synthesis studies [75–77]. Those rules are very high cooling rates, the presence of at least three constituting elements, atomic size differences over 12%, a significant negative heat of mixing of major elements, small material dimensions (preferably at the nanoscale, favoring high cooling rates), and post-processing effects. Furthermore, metalloid elements, such as phosphorous, silicon, boron, and carbon were shown to retard crystallization and favor the formation of long-range disordered structures. Organic solvent molecules used in LSPC can serve as a carbon source which strongly affects the stabilization of amorphous structures while the influence of the other mentioned metalloids is less frequently observed [75]. Crystalline FeSiB NPs, synthesized by ns-LAL in water could be modified and an amorphous structure could be achieved by a follow-up LFL treatment. As a result, the particle size decreased and the beforehand dominant α-Fe structure could not be observed after LFL, and solely amorphous structures were found [76]. Ps-LAL of FeSiB in organic solvents, however, yielded amorphous NPs directly after ablation in acetone, ethanol, and acetonitrile, while nanocrystalline byproducts of α-Fe/Fe_3_C phases were also detected after synthesis in ethanol [77]. Recently, Su et al. showed amorphization of wet-chemically synthesized crystalline FeNi NPs encapsulated in N-doped carbon nanotubes by re-irradiating the samples with a pulsed laser beam [78]. In brief, amorphous alloy NPs by laser-based synthesis techniques are not well studied, though representing an emerging field due to applicability in catalysis. In particular, amorphization of HEA NPs from LSPC has only been reported in one study [34] and it is unknown which factors determine whether the produced particles are crystalline or amorphous.

Herein, we report on the laser synthesis of colloidal Cantor alloy (CrMnFeCoNi) NPs, systematically varying pulse duration (ps and ns pulses) in laser ablation in organic solvents (acetone, ethanol, acetonitrile), aiming to deduce general design rules triggering the formation of crystalline or amorphous structures in the corresponding particles. All three organic solvents were chosen to ensure comparability with prior studies on HEA NPs, as well as other alloy systems (CoAu [68], FeAu [79]), which were produced by laser ablation in organic liquids, and are technical solvents used in the chemical industry. Nanoparticle characterization is done by high-resolution transmission electron microscopy (HRTEM), energy-dispersive X-ray spectroscopy (STEM-EDX), selected-area electron diffraction (SAED), X-ray diffraction (XRD), and electron energy loss spectroscopy (EELS), complemented by tempering and laser post-irradiation experiments to pinpoint HEA NP formation pathways into thermodynamically favored or kinetically stabilized structures.

## Results and Discussion

Picosecond-LAL (wavelength: 1064 nm, pulse energy: 0.15 mJ, repetition rate: 100 kHz, incident fluence: 0.1 J/cm^2^) and ns-LAL (wavelength: 1064 nm, pulse energy: 0.50 mJ, repetition rate: 10 kHz, incident fluence: 7 J/cm^2^) of the bulk target in all three solvents yielded colloids at mass concentrations of 100–200 mg/L, determined by differential weighing of the target before and after ablation. The total composition of the colloids in acetone were determined by STEM-EDX and were similar for both laser pulse durations, namely Cr_22_Mn_17_Fe_21_Co_20_Ni_20_ for colloids made by ps-LAL and Cr_21_Mn_21_Fe_20_Co_19_Ni_19_ for colloids fabricated by ns-LAL (global values determined via STEM-EDX displayed in Supporting Information File 1, Table S1 and Table S2). These values are in good agreement with the near-equimolar composition of the bulk target (Cr_21_Mn_18_Fe_21_Co_18_Ni_22_), determined by both XRF and SEM-EDX measurements, quantifying not only the surface compositions of the corresponding targets but also bulk compositions determined from a target cross-section (Supporting Information File 1, Figure S2).

### Pulse duration effects on the crystal structure and composition

**Figure 1:**
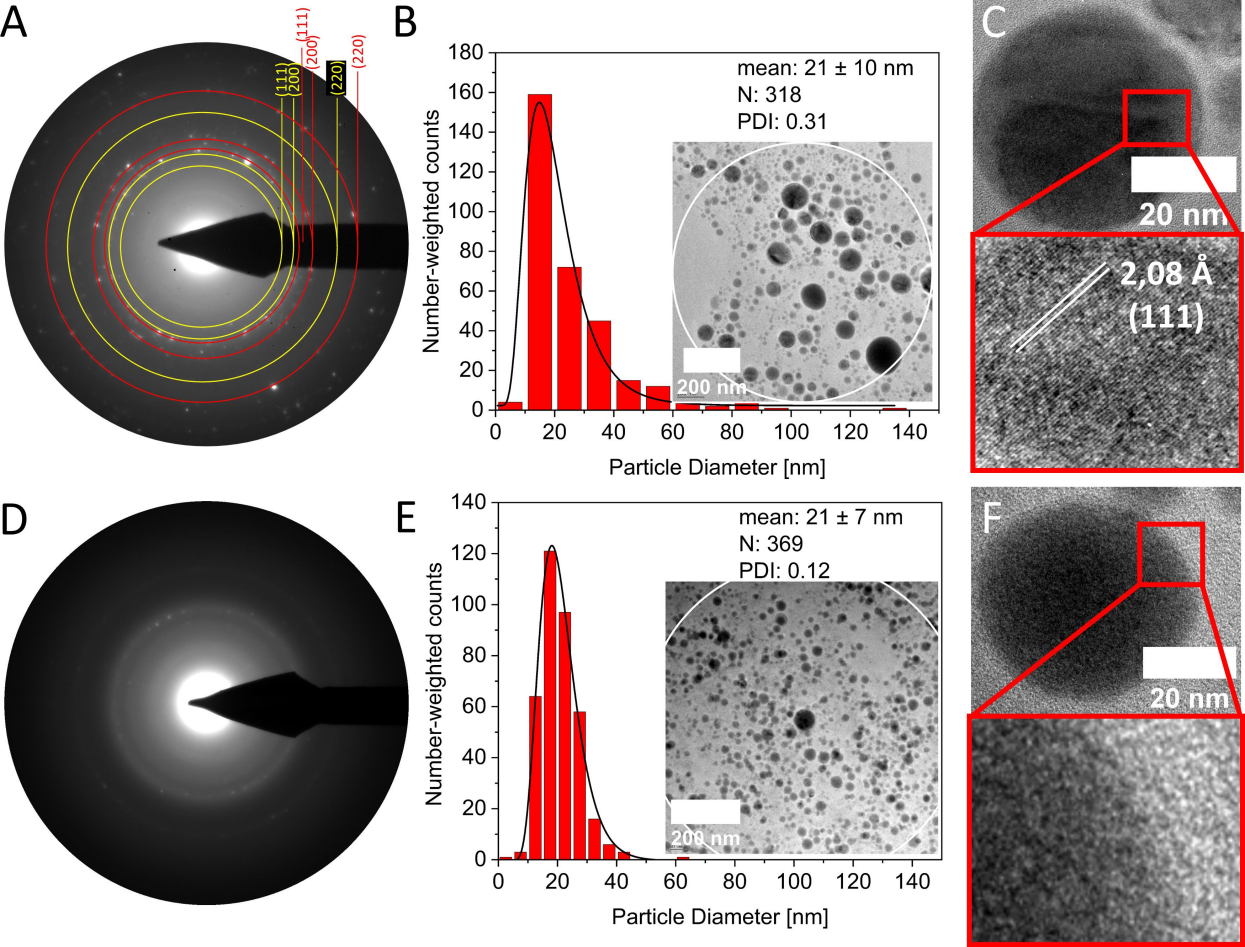
SAED pattern of ps-ablated crystalline HEA NPs in acetone (red: reflections assigned to fcc and yellow: reflections assigned to MnO) (A) and the corresponding analyzed area with particle size distribution with a mean diameter of 21 ± 10 nm (B) and ns-ablated amorphous HEA NPs (D), also with its corresponding analyzed area as well as particle size distribution with a mean diameter of 21 ± 7 nm (E). Please note that all SAED data are based on particle ensembles, and the exemplary images analyzed with corresponding size distributions, mean diameters, number of particles (N), and PDI values are displayed. (C) Depicts an HRTEM micrograph of a crystalline HEA NP, made by ps-LAL, representing an fcc structure with a *d*_(111)_-value of 2.08 Å and (F) shows an amorphous particle, by ns-LAL, exhibiting no structural motif.

The first objective of this study was to investigate the influence of pulse durations during LAL in acetone (dried, distilled, and degassed) on the crystal structure and composition of the corresponding HEA NPs. Figure 1 shows a representative result of colloids from LAL in acetone analyzed by SAED on ensembles of 300–400 HEA NPs. These findings highlight the formation of crystalline particles with fcc structure and estimated *d*-values of 2.09 Å (111), 1.83 Å (200), and 1.29 Å (220) during ps-LAL (Figure 1A). This is in good agreement with former ps-LAL studies by Waag et al. and also by Löffler et al., who reported comparable *d*-values of 2.08 Å (111), 1.81 Å (200), and 1.28 Å (220), determined by both SAED and XRD analysis [18,35]. Conversely, amorphous structures were found during ns-LAL (Figure 1D), showing no Bragg reflections. This observation was also confirmed by HRTEM micrographs of representative particles where a (111)-lattice spacing with a *d*-value of 2.08 Å (Figure 1C) was observable for NPs from ps-LAL, being in good agreement with the estimated value in the bulk target (*d*_(111)_-value of 2.08 Å, compare with Supporting Information File 1, Figure S2). In contrast, Figure 1F shows the HRTEM micrographs of an amorphous particle, resulting from ns-LAL in acetone. As the number of analyzed particles is similar in the SAED analysis data from Figure 1A and Figure 1D, a dependency of the crystal structure in ignoble HEA NPs on pulse duration (ps vs ns) is hypothesized. To further investigate this hypothesis, we looked at further potential influence factors, such as a potential dependency on particle sizes, solvent type, deviating compositions in the ablated bulk targets and HEA NPs, laser fluence, and post-irradiation effects.

One assumption may be that amorphous phase formation is ruled by the particle diameter as it is well-known that smaller NPs may appear amorphous in diffraction-based techniques due to less coherent conditions for Bragg reflection from their crystalline domains [80–81]. To test this hypothesis, we analyzed the particle size distributions within the circular areas used for SAED experiments for amorphous samples from ns-LAL and crystalline samples from ps-LAL. Figure 1B and Figure 1E show TEM images of analyzed HEA NPs with the corresponding particle size distributions directly associated with the diffraction patterns of the SAED analysis. In both selected areas (indicated as white circles in Figure 1B and Figure 1E, respectively) the determined particle size distributions show mean values of 21 ± 10 nm for crystalline ps-ablated particles and 21 ± 7 nm for amorphous ns-ablated particles. Based on these findings we can conclude that the pronounced differences in crystallinity in the samples cannot be explained by differences in particle diameters. However, a certain width of the size distribution curves indicated by the polydispersity indices (PDI) (PDI = 0.31 for ps-LAL HEA NPs and PDI = 0.12 for ns-LAL HEA NPs) is evident. Due to fundamental differences in the particle formation mechanisms during ps- and ns-LAL as proposed by Shih et al., ps-LAL has been reported to yield bimodal size distributions (at least of Ag and Au at high fluences) [82], which may be the reason for the broader size distributions in ps-LAL. Note that Waag et al. reported mean particle sizes of <10 nm determined by both centrifugation-based methods as well as TEM during ps-LAL of equimolar CrMnFeCoNi in ethanol [35], whereas in our work we observed mean diameters of *d*_mean_ = 21 ± 10 nm after ps-LAL in acetone. As in their work different pulse energies were applied (88 µJ vs 150 µJ in our work) and a different setup was utilized (flow-through chamber vs semi-batch chamber in our work), it is likely that in our case the ablation process happens under harsher conditions. The difference in pulse energies possibly leads to stronger spallation [50,83] and thereby result in larger HEA NPs. Further, we cannot exclude that ripening processes occur in the semi-batch chamber utilized in this work, which would be less likely for the flow-through chamber used by Waag et al., as here the formed nanoparticles do not tend to accumulate and local nanoparticle concentrations are lower. However, to verify that the mentioned size difference can be transferred to the investigated organic solvents in this work, we compared mean size diameters of HEA NPs synthesized under similar conditions in Supporting Information File 1, Figure S3 and Figure S5. We concluded that due to the similarity in mean size, this observed size difference can be analogously observed for the synthesis in acetone, ethanol, and acetonitrile.

The similarity in particle sizes of Figure 1B and Figure 1E with the absence of SAED reflections in ns-LAL HEA NPs indicate that the results are independent of the signal-dominant mean particle size of the sample.

### Solvent type variation

To test whether amorphous phase formation is, furthermore, applicable to the solvents used, we conducted additional ns-LAL in ethanol and ps-LAL in acetonitrile, complementary to studies previously reported by Waag et al. [35] (ps-LAL in ethanol for CrMnFeCoNi) and Johny et al. [34] (ns-LAL in acetonitrile also for CrMnFeCoNi). The corresponding data are presented in Supporting Information File 1 (Figure S3) and clearly show that the hypothesized pulse duration-driven effect is applicable to all three tested solvents acetone, ethanol, and acetonitrile. In all these solvents ns-LAL leaded to amorphous NPs while during ps-LAL the particles were crystalline.

### Crystallography and (traces of) oxidation

**Figure 2:**
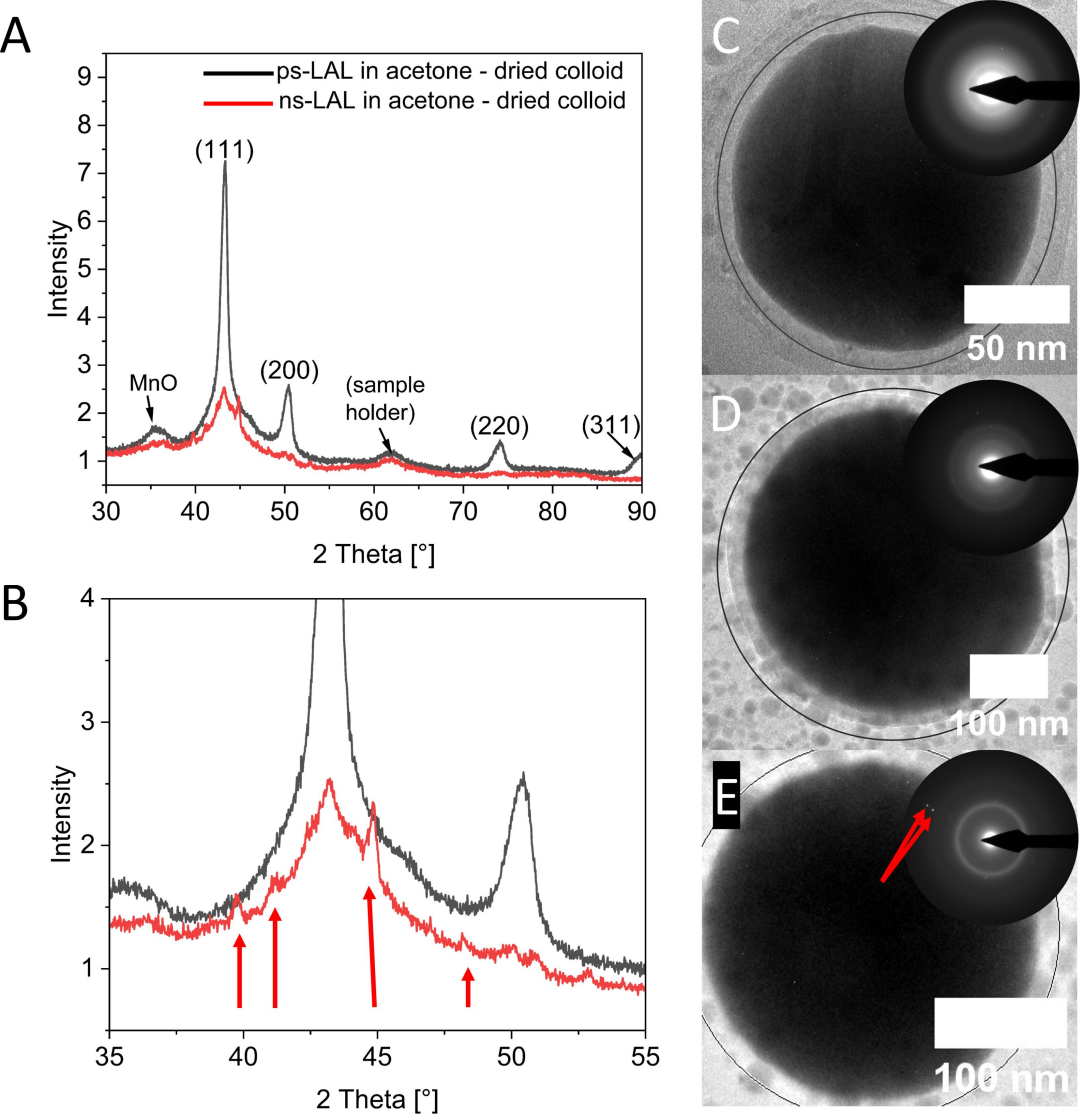
X-ray diffraction patterns of ps-LAL and ns-LAL HEA NPs, indicating a decrease in crystallinity in ns-LAL HEA NPs (A) and a magnified area (B) with arrows depicting unknown phases with relatively sharp reflections of crystallite sizes between 40–50 nm in ns-LAL HEA NPs. (C, D, E) Selection of particles by ns-LAL with diameters above 100 nm and the single-particle SAED as an insert, showing that big particles are formed fully amorphous (C, D) or with minor crystalline reflections (indicated by red arrows) (E). Grey circles in C–E indicate the analyzed area for single-particle SAED analysis.

Further investigations on HEA NPs are required to determine whether the structural information obtained from Figure 1 can also be reproduced for a larger fraction of the samples. Thus, XRD measurements were conducted on both ps- and ns-ablated HEA NPs. Figure 2 shows representative diffraction patterns of ps-ablated HEA NPs and ns-ablated HEA NPs (Figure 2A), both synthesized in acetone. The particles from ps-LAL show a distinctive crystalline fcc diffraction pattern of CrMnFeCoNi NPs reflections at the diffraction angles 2θ = 43.3°, 50.5°, and 74.1° and the corresponding lattice parameter of 3.635 Å as determined by Rietveld refinement, which agrees well with the lattice parameters of the bulk target (3.601 Å) (Supporting Information File 1, Figure S2) and those from previous studies [35]. Note that the small difference in the lattice constants between NPs and the bulk could be attributed to lattice strain in the particles resulting from rapid heating and cooling processes with rates of up to 10^13^ K/s [50] during LAL. An average crystallite size of 10 nm was determined, which indicates the presence of polycrystalline HEA NPs. Additionally, a broad (110)-intensity of manganese(II)-oxide can be detected at 35.6° with a crystallite size calculated to be 3 nm. Thin oxide shells, mainly formed by manganese oxide species, can be found in STEM and TEM images from ps- and ns- synthesized NPs (compare with Supporting Information File 1, Figure S4) and could be one explanation for the oxide phase detected in the XRD analysis of Figure 2A. Note that aside from oxide shells, carbon shells were also found in some of the observed HEA NPs in both ns-LAL and ps-LAL in acetone (exemplary shown in Supporting Information File 1, Figure S4). In general, not all HEA NPs were found to possess oxide or carbon shells resolvable by TEM. Compared to the results from Johny et al. who synthesized HEA NPs in acetonitrile by ns-LAL and observed prominent carbon shells [34], the thickness of the carbon shells after ps- and ns-LAL in acetone is significantly smaller. Therefore, we can conclude that LAL in acetone yields both oxide and carbon-encapsulated HEA NPs while latter was found to vary between minimal, almost non-observable carbon shell and well-visible shell thickness (3–4 Å) in TEM.

**Figure 3:**
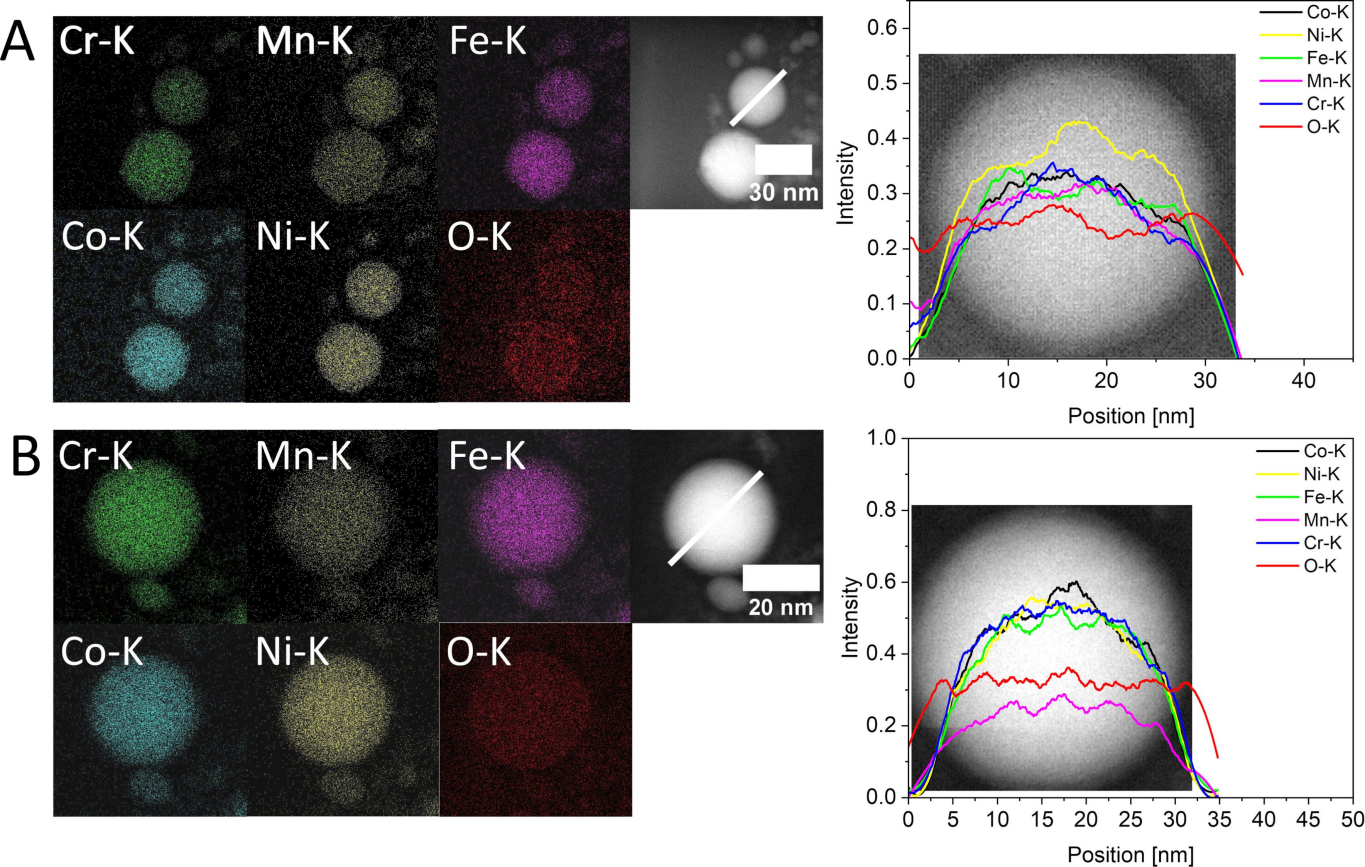
STEM-EDX characterization of HEA NPs by ps-LAL (A) and ns-LAL (B) with EDX maps of Cr, Mn, Fe, Co, Ni, and O, all signals extracted from K-shell and additional line scans of chosen particles. The positions of the line scans are both marked in the STEM images to the corresponding EDX maps. All elements are homogeneously mixed within the particles and show no qualitative differences between the two synthesis conditions.

In a direct comparison of the X-ray diffraction patterns in Figure 2 (ns vs ps), the diffraction pattern of ns-LAL HEA NPs shows a significant decrease in crystallinity (Figure 2A, red line). As comparable masses of NPs were analyzed in each XRD analysis, it is not relatable to a lack of X-ray intensity from the sample volume. Even though the diffraction patterns of ns-LAL HEA NPs confirm that mainly amorphous HEA NPs are present, a small portion of crystalline structures contribute to the (111)-reflection of the fcc structure (average crystallite size = 10 nm). This follows a study by Johny et al., where the authors also reported a small amount of larger crystalline particles after ns-LAL of Cantor alloy NPs in the sample by XRD, among the dominant amount of amorphous particles [34]. Additionally, a few sharper reflections from unknown phases, having crystallite sizes between 40 to 50 nm (after anisotropic refinement) could be detected (marked by red arrows in Figure 2B), but due to their weak intensity, an identification was not possible. The presence of those unidentified reflections is most likely due to the formation of minor mass fractions of HEA NPs, exceeding a particle diameter of several hundreds of nanometers, which are partially crystalline. To selectively analyze the larger particle fractions with diameters >100 nm, single-particle SAED analysis was conducted (Figure 2D–F). Interestingly, structural heterogeneity was observable at this size regime. While the first two particles are still fully amorphous and thus strengthen our hypothesis that amorphization is not linked to particle size, the particle in Figure 3F shows two reflections, although they do not result from a metallic fcc structure. With *d*-values of 1.09 Å and 0.98 Å, they are most likely attributed to multiple-element oxides such as (FeMn)O. As such species cannot be identified in the diffractogram, we can conclude that those oxides are not formed in the majority of the HEA NPs. Based on these complementary findings from XRD and single-particle SAED, we can summarize that reflections found in the otherwise amorphous samples are not attributed to a size-dependent difference in crystal structures but are probably derived from minor crystalline oxide phases. To further highlight that amorphous phase formation after ns-LAL is an analogous effect in other solvents, XRD analysis was also conducted on particles synthesized in ethanol (see Supporting Information File 1, Figure S5). Here, the difference in phase formation appears analogously to the HEA NPs in acetone, and particles via ps-LAL form crystalline structures while their counterparts via ns-LAL form mainly amorphous structures. Note that analogously to the ns-LAL-generated HEA NPs in acetone (Figure 2A and Figure 2B), a small contribution to a crystalline reflection is also observable, stating that a minor fraction of crystalline HEA NPs still forms, although the majority of the sample is found to show an amorphous phase. Comparing the observed reflections in single-particle SAED with the unknown reflections in the X-ray diffractogram of Figure 2A and Figure 2B, we see that the unknown reflections most likely result from oxide phases, although the minor contribution of the (111)-reflection from XRD could not be found in the larger HEA NPs by ns-LAL in single-particle SAED. Thus, we cannot fully exclude the presence of a minor fraction of crystalline particles during ns-LAL although the vast majority is confirmed to be amorphous.

As different pulse durations (ps vs ns) may lead to fundamentally different ablation mechanisms during LAL [50,64], it is conceivable that differences in sample compositions (content and distribution of the elements Cr, Mn, Fe, Co, Ni) may be responsible for the observed pulse-duration-dependent differences in crystal structures. To examine this issue, we initially analyzed the crystal structure of the bulk target via XRD (Supporting Information File 1, Figure S2). The structure of the target is characterized by two fcc structures with lattice constants of 3.601 and 3.595 Å with average crystallite sizes of 108 and 153 nm, respectively. Furthermore, the elemental composition of the target was analyzed by SEM-EDX and XRF, differentiating compositions on the upper and lower surfaces of the target as well as the core composition determined by EDX after target slicing. The overall composition of the elements in the target is fully homogeneous though it should be noted that the diameter of the used SEM-EDX probe beam is 1 µm. Thus, potential differences in the composition between the different crystallites with average diameters of 108 and 153 nm may not have been resolved. So, it is conceivable that small differences in the composition of the crystallites may have been responsible for the occurrence of two fcc phases. The determined values considering crystallite size and lattice constants by XRD agree with former studies on this element system in the bulk state [84]. In consecutive experiments, we also analyzed the elemental composition of the targets after ps-LAL and ns-LAL using EDX. The findings show that the surface retains its homogeneous distribution of elements and no favored ablation of single elements with resulting element islands from non-ablated elements can be observed. Thus, besides some topographic differences visible at high magnifications due to the target surface melting process in ns-LAL, only minor compositional differences (3–4 atom %) are observed. Those, however, are in line with general deviations of resulting HEA NPs and expected statistical errors in EDX analysis (±2% to ±5%), making it unlikely that those differences will have a big impact on structural differences. Based on this data, we can conclude that element ratios close to the mole fraction of the target are converted into NPs (Supporting Information File 1, Figure S6).

### High-resolution particle characterization

The elemental distribution on a single-particle level may still be different between NPs from ns- and ps-LAL. To this end, the formed HEA NPs were examined by STEM-EDX analysis. A homogeneous element distribution confirmed by EDX maps and representative EDX line scans is observed for both crystalline particles by ps-LAL (Figure 3A), as well as amorphous particles by ns-LAL (Figure 3B). The oxygen signals, equally pronounced in NPs from ps- and ns-LAL hint towards oxidation of the particles. Even though these measurements cannot differentiate between an oxidized surface and an oxidized core of the particle, its horizontal progression of oxygen signal hints towards surface oxidation. This follows the XRD results (Figure 2A and Figure 2B) where reflections attributed to MnO were identified, most likely resulting from thin oxide layers on top of metallic HEA NPs as can be seen in Supporting Information File 1, Figure S4. Oxidation of HEA nanoparticles was already reported by Johny et al. and also Tahir et al., including atom probe tomography analysis, showing that oxidation processes in LAL in organic liquids cannot be completely prevented (but at least reduced down to a few atom %) [34,63]. In some cases, a depletion of Mn is seen in exemplary HEA NPs of different sizes (ranging from 20–160 nm in diameter) when the composition of single particles is analyzed (see Supporting Information File 1, Tables S3 and S4, Section S8). Independent of size, the deviations can differ by more than a factor of two in manganese content. Deviating manganese contents were already observed in the works by Johny et al. [34] and Tahir et al. [63] and depletions can be explained with manganese having the lowest melting point and highest vapor pressure [85–86], but also due to its negative redox potential and high oxygen affinity that favors Mn leaching based on chemical mechanisms [19]. Further, due to the small crystallite size of 3 nm, calculated from the Mn oxide reflections in both ps-HEA NPs and ns-HEA NPs (Figure 3A and Figure 3B), the existence of small MnO precipitates is also possible, which may explain the lack of Mn in individual HEA NPs, while global EDX compositions are comparably close to the bulk target and are in line with minor deviations in both dried colloid and bulk target. However, despite some exceptions as seen in Supporting Information File 1; Table S4, most HEA NPs are Mn-depleted, and MnO precipitates probably account for the compositional differences between single particles and larger ensembles. Some HEA NPs after ns-LAL show slightly increased values in Mn-content (Supporting Information File 1, Table S4). Even though these values are within the margin of statistical errors in EDX (±2% to ±5%) they may be related to the known limited lateral elemental mixing of alloy targets during LAL [87]. It is conceivable that Mn, as the metal with the lowest melting point within the HEA, solidifies last after melting and is enriched in the melted area of the target. As this target remelting already occurs after the first laser pulse, continuous ablation of the same area with consecutive pulses could lead to the creation of NPs with Mn enrichment.

All in all, the composition of metallic constituents in all HEA NPs analyzed agrees well with the determined composition of the bulk target which was conducted by both EDX and XRF analysis (Supporting Information File 1, Figure S2, Section S2, and Section S8).

Note that LAL experiments to fabricate the HEA NPs in this work were conducted using lasers with strongly deviating pulse energies (ps-LAL: 0.15 mJ; ns-LAL 0.50 mJ), laser fluences (ps-LAL 0.1 J/cm^2^; ns-LAL 7 J/cm^2^), and repetition rates (ps-LAL 100 kHz; ns-LAL 10 kHz). To verify that the varied parameters in our study do not influence amorphization, we conducted control experiments and applied the same laser fluences in the first control experiment (1.6 J/cm^2^) and the same pulse energies in the second control experiment (0.15 mJ) (corresponding parameters can be found in Supporting Information File 1, Table S5, Section S9), exceeding typical threshold values by far [50,88]. As depicted in Supporting Information File 1, Figure S7, amorphization tendencies were analogous in all control experiments, accounting for both laser fluence and pulse energy. Since repetition rates vary by a factor of ten, it appears reasonable that this may also affect particle-forming mechanisms. However, various studies show that particles already form after a few nanoseconds [50,64,82]. As we applied repetition rates of 100 kHz (ps-LAL) and 10 kHz (ns-LAL) in this study, a second pulse hits the target surface with a delay of 10 µs and 100 µs. Hence, particle formation timescales are orders of magnitude shorter than the inter-pulse delay, indicating that the impact of the repetition rate on particle formation is negligible.

### Post-irradiation control experiments

**Figure 4:**
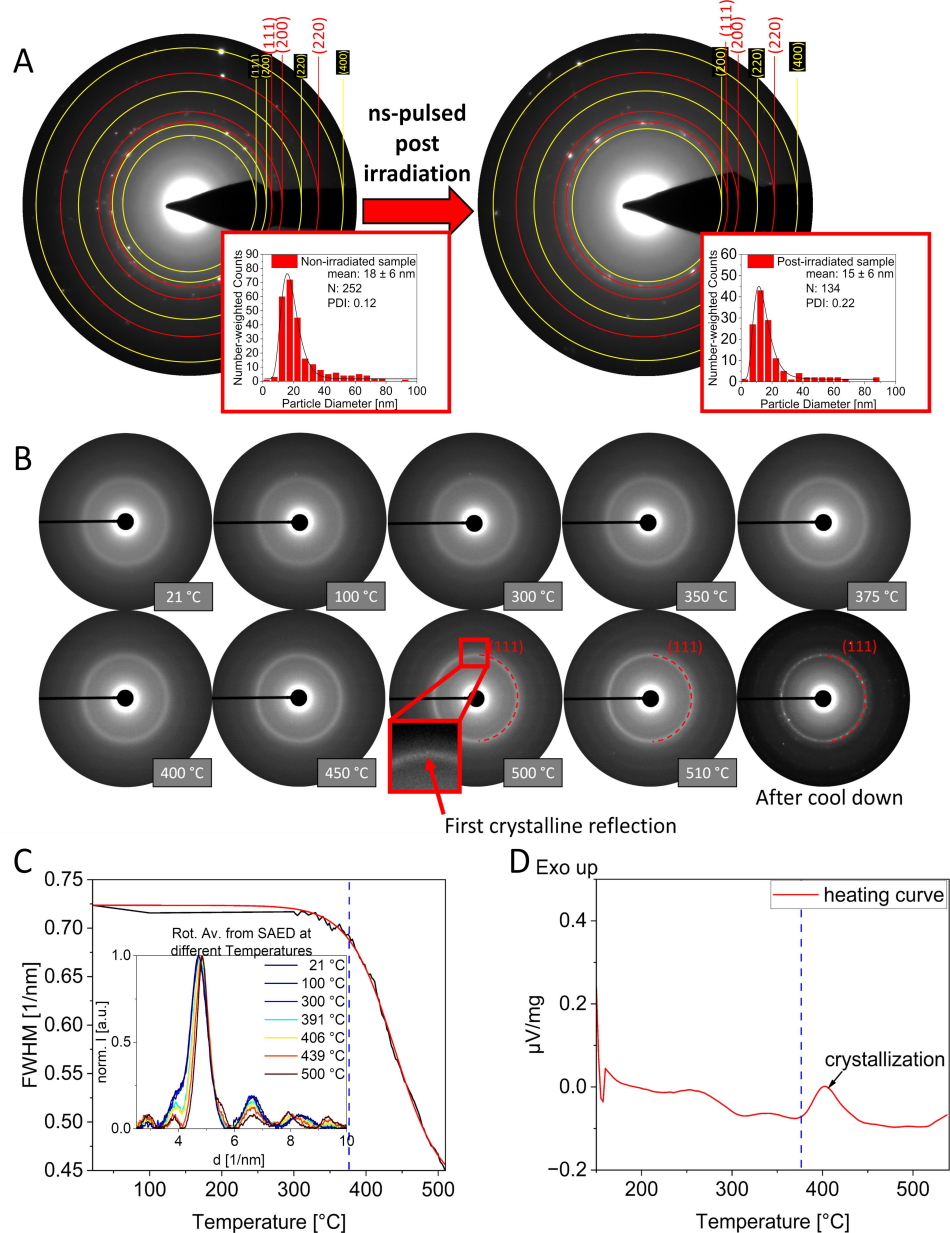
(A) Post-irradiation experiment of a colloid obtained by ps-LAL (left), showing both the SAED pattern and particle size distribution (with mean diameter, number of particles (N), and PDI), and the colloid obtained after irradiation with ns-pulses (right) with no decrease in crystallinity and no significant difference in the particle size distribution. Here, reflections displayed in red are assigned to the fcc phase whereas reflections marked with yellow circles are derived from metal oxides. (B) In situ TEM heating experiment of amorphous HEA NPs by ns-LAL, depicting SAED pattern in different temperature steps with a first reflection ((111)-fcc) at 500 °C. After cooling down, a significant intensity of reflections is detectable. (C) FWHM of the (111)-fcc rings (derived from rotational average) in the SAED patterns of the heating experiment show a significant decrease starting at around 375 °C, underlining the starting crystallization of the amorphous particles. The black line shows raw data and the red line represents a Boltzmann fit. (D) DSC heating profile of the amorphous HEA NPs obtained by ns-LAL shows the exothermic crystallization process that begins at 375 °C, complementing the heating experiments and the analysis of the FWHM.

Based on these experimental series we can conclude that the pulse duration during LAL synthesis of CrMnFeCoNi HEA NPs is the main discriminator driving the prevalence of amorphous or crystalline phase structures. But how does the pulse duration affect the structure of the particle? To resolve this conundrum, we first need to clarify whether amorphization is a phenomenon that directly occurs during particle formation or whether post-irradiation effects are involved. As stated in the experimental section, LAL was conducted in a stirred semi-batch ablation chamber described elsewhere [89] whose constructional design is shown in Supporting Information File 1, Figure S1. A part of the stirred ablation liquid is constantly in front of the bulk target and is thus constantly irradiated by the pulsed laser beam. Consequently, the number of laser pulses per particle changes with ablation time. A previous study by Su et al. shows amorphization of nanoparticles after laser post-processing [78], indicating a potential interplay between amorphization and post-irradiation. To understand whether the amorphous phase formation in ns-LAL HEA NPs is a result of post-irradiation, freshly formed crystalline particles, the crystalline HEA NPs made by ps-LAL in acetone, were irradiated with ns-pulses at a wavelength of 1064 nm. We also checked whether amorphous NPs derived from ns-LAL could be crystallized by post-irradiation with ps-pulses. As can be seen in Figure 4A and Supporting Information File 1, Figure S8, the results show that post-irradiating HEA NPs does not affect the crystallinity of the particles in both cases. To highlight this, Figure 4A shows the electron diffraction patterns and number-weighted size distributions of HEA NPs by ps- LAL (left) and the particles after irradiating with ns-pulses (right). Interestingly, the crystallinity of the particles does not change after irradiation, and the fcc structure is stable against the irradiation with *d*-values of 2.09 Å (111), 1.83 Å (200), and 1.29 Å (220) (marked red) and additional reflections mainly resulting from MnO but also from other potential unknown oxide species (marked yellow). This diffraction pattern is thus comparable with the pattern of the particles before the experiment (Figure 4A left), having the same *d*-values for the fcc structure. Consequently, post-irradiation experiments, linked to repetition rates, show that neither post-irradiation of formed NPs nor target surface affect the final structure of HEA NPs. It is important to note that the structural differences arising from the application of the two different pulse durations did not occur coincidentally but are a reproducible observation (shown in Supporting Information File 1, Section S11).

### Thermal stability of the amorphous HEA nanoparticles

Finally, we aimed to examine the temperature stability of the amorphous HEA NPs. Thereto, we conducted in situ heating experiments in TEM as well as differential scanning calorimetry. Figure 4B displays the TEM in situ heating experiment and SAED patterns of an amorphous sample made by ns-LAL in acetone with increasing temperature. The diffuse and broad intensity that results from an amorphous structure gets sharper, especially for the (111)-ring, meaning that the energy input by heating results in the formation of crystallites which grow with increasing temperatures. At 500 °C the first weak reflection appears, with a *d*-value of 2.1 Å which belongs to the (111)-face of the fcc-structure (compare with Figure 1A and Figure 2A). These reflections get more pronounced after cooling down, which can be attributed to enhanced Bragg diffraction at lower temperatures while crystallite growth after particle cooldown could also be possible. This is consistent with the heating providing the activation energy for the initiation of this process followed by spontaneous transformation into the thermodynamically favored crystalline state. In addition, we observe a decrease of the full width at half-maximum (FWHM, derived by rotational average from the SAED analysis) of the investigated (111)-ring in the in situ heating experiment (Figure 4C), which indicates the formation of larger crystallites in SAED. As shown in the differential scanning calorimetry (DSC) measurement in Figure 4D, the crystallization of the amorphous particles is an exothermic process and begins at approximately 375 °C, which is exactly the same temperature where the rapid decrease of the FWHM in Figure 4C starts, and the required thermal energy *k*_B_T is achieved to overcome the activation energy. Note that for the determination of the plot in Figure 4C, more temperature steps were considered than those shown in Figure 4B. However, for clarity reasons, the full data set of 94 SAED patterns is not shown and can be found in the Supporting Information File 2 as a video file. The observation of amorphous particles transforming into a crystalline phase validates that the amorphous phase is metastable and forms under kinetic control, whereas the crystalline structure is the thermodynamically favored one [90–91], and thermodynamic control is dominant when activation energy barriers are overcome by heating.

### Mechanistic discussion

Based on these findings we can conclude that the crystal structure of these HEA NPs is pre-determined directly during particle formation, probably in the ablation plume phase. Previous studies investigated the LAL process using a picosecond-pulsed laser in comparison to a nanosecond-pulsed laser [50,64,82]. As stated in these works, ps-LAL leads to more vigorous ablation conditions (up to 12000 K maximum temperature and peak pressures of up to 38 GPa), related to the conditions of stress and thermal confinement of the deposited laser energy. Conversely, in ns-LAL the energy of the laser pulse is transferred deeper into the bulk target, and peak temperature and pressure are significantly lower (about 5000–8000 K and 4.8 GPa). It is important to note that the difference in pressure was shown to not affect the formation and crystallization of the particles but rather dictates the presence or absence of thermal and stress confinement during ps- and ns-LAL and, thus, plays a crucial role in the heating and mixing behavior of the ablation plume [64]. Also, those numbers as well result from a computational model of pure silver and FeNi in water for ps-LAL and ns-LAL; therefore, transferability to HEA NPs in organic solvents may be limited. Additionally, the longest calculated pulse in [64] has a length of 2 ns, whereas in this work the pulse duration is 10 ns in the case of ns-LAL, possibly leading to conditions that may be even more dictated by thermal processes. Consequently, we can expect a longer heating of the plume in our experiments than that observed in the simulated study, and thus longer heated periods during the early phase of LAL. Although the aforementioned studies describe different processes that happen during LAL with different pulse durations, the factor with the most pronounced impact on amorphization in this context could be the particle cooling rate. However, the cooling rates during ps-LAL and ns-LAL are estimated to be both high enough to initiate amorphization, but are also in a comparable regime with rates of 10^12^–10^13^ K/s in both cases [50,64]. Based on this, we can conclude that the pronounced differences in amorphization during ps-LAL and ns-LAL are not caused by differences in cooling rate, but by the different plume heating times during LAL. Moreover, it is unlikely that the heating duration is the only prerequisite for amorphous phase formation, but also the different intensity and/or duration of chemical reactions of the plume with the solvent could contribute.

**Figure 5:**
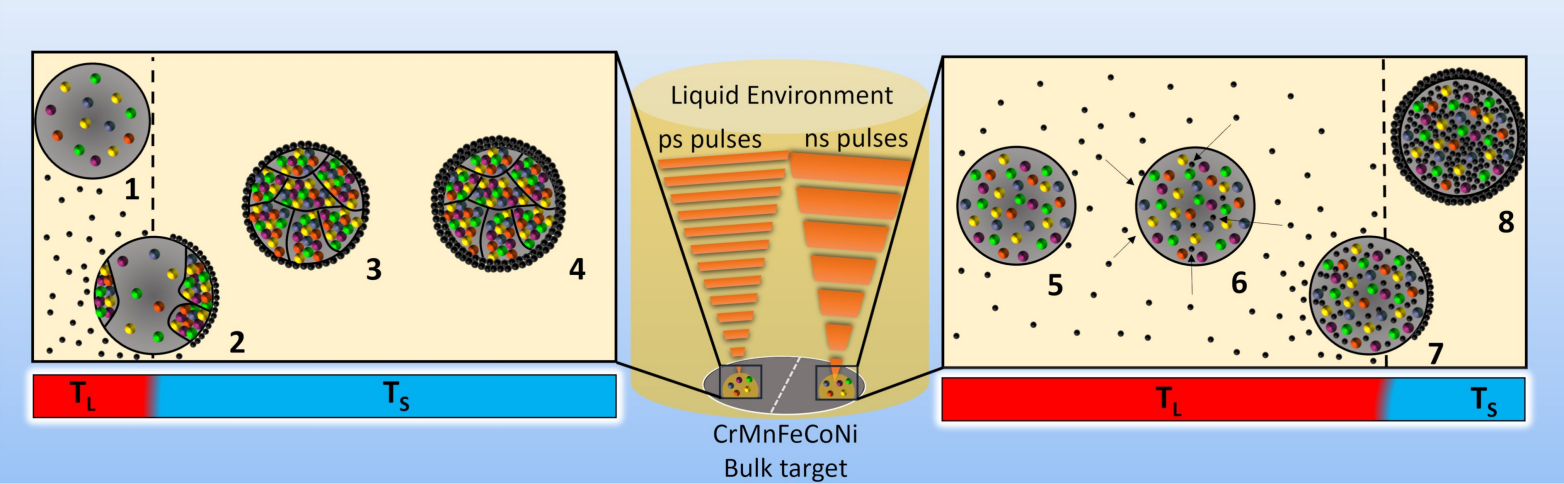
How the particle structure is set by laser pulse duration: Graphical illustration of the hypothesized mechanism behind the pulse-dependent structure of CrMnFeCoNi HEA NPs by ns- and ps-LAL in organic liquids, stating the interplay between metallic atoms (colored spheres) and active carbon species (black spheres). In the center, the bulk material is ablated by either nanosecond or picosecond pulses in liquid (the liquid is illustrated by a yellow cylinder). During picosecond LAL, after the energy transfer into the bulk target, the plume with liquid nanodroplets forms (at liquid-state temperatures, *T*_L_), surrounded by active carbon species (1). Solidification temperatures (*T*_S_) are reached rapidly (2) which yields (poly)crystalline particles with carbon shells (3) which possibly can grow due to post-condensation processes (4) (similar post-condensation mechanisms were also postulated and discussed by Fromme et al. in reference [93]). Conversely, during nanosecond LAL, the period of *T*_L_ lasts significantly longer as the energy from the pulse is pumped into the plume three orders of magnitude longer. Consequently, the particles stemming from the plume which is in a liquid state (5) can be doped more intensively by diffusing carbon into the molten metal core (6 and 7). After solidification, the NPs cannot form proper crystallites with the expected fcc structure, but form C-doped amorphous structures, analogously surrounded by carbon shells as well (8).

One difference between LAL with ps and ns pulses is that ns-LAL goes along with a more pronounced melting of the target surface (higher thermal penetration depth and lateral heating), compared to that of ps-LAL, a process that Waag et al. experimentally described by comparing target surfaces after both ps- and ns-LAL [87]. This is because in ns-LAL, thermal processes are dominant and thus, the ablation process is not as vigorous as in ps-LAL. Further, due to the longer exposure of thermal penetration in ns-LAL, the ablation plume tends to be heated for a longer time compared to that in ps-LAL. However, in both ns- and ps-laser ablation in organic solvents, active carbon species (radicals, permanent gases, degradation and condensation products as well as elemental carbon) were shown to form by various studies, conducting techniques such as gas-displacement quantifications and subsequent analysis via gas chromatography-coupled techniques [92–94] while a recent review also thoroughly reported on the by-product development due to radical recombination during laser-based syntheses in organic solvents [54]. These carbon species can interact with the plume and diffuse into the nanoparticles, depending on the affinity of nanoparticle constituting elements to carbon or carbon solubility [40,95–96]. Note that the degradation of solvent molecules is even higher for ps-LAL than for ns-LAL [54], meaning that the resulting structural differences in HEA NPs do not occur due to a higher availability of carbon. Diffusing carbon into a metallic matrix has been reported for monometallic systems [95,97–98] and alloys [76–77], stabilizing amorphous structures and enhancing glass formation ability (GFA). In general, Si, B, P, and carbon have been reported to diffuse into metallic matrices and thus enhance the GFA, whereas carbon seems to have the biggest effect on GFA [98], thus resulting in an amorphous structure with short-range order [75]. As ns-LAL heats the plume orders of magnitude longer than ps-LAL, we hypothesize that the organic solvent molecules and the formed carbon species may react longer at the plume front, particularly in the mixing region where the ablated matter interpenetrates the solvent vapor. In that region, the nanoparticle droplets stemming from the plume cool fast and solidify first [50]. Consequently, the carbon species will have more time to interact with the plume and diffuse more significantly into the metallic matrix of the forming HEA NPs, being the reason behind the stabilized amorphous structure. Figure 5 illustrates the hypothesized differences during ps- and ns-LAL. In ps-LAL, the time when the temperature is high enough for the plume and particles to stay in a liquid state (*T*_L_) is short and can be expected to be only a few ns (pulse duration of 10 ps and additional heated period [50]). The molten particle that is surrounded by reactive carbon species (Figure 5 (1)) is rapidly cooled down by heat exchange with the solvent and experiences a fast transition down to temperatures that are low enough for undercooling and solidification (*T*_S_) (Figure 5 (2)) whereas the carbon (if a carbon shell is formed) rather stays on the surface of the particle. Consequently, the HEA NPs are formed in crystalline nature (single- or polycrystalline, often with defects) with potential carbon species on their surfaces (e.g., graphitic carbon shells) (Figure 5 (3) and (4)). In contrast, during ns-LAL, the conditions of *T*_L_ are present for a longer time and thereby the plume and alloy droplets are expected to stay in these conditions for approximately 15 ns (pulse duration of 10 ns and additional heated period [64]). Consequently, the emerging liquid nanoparticles (Figure 5 (5)) are in a liquid state for a longer time. Thus, active carbon species can diffuse more significantly into the molten metal core of the nanoparticles (Figure 5 (6)), although a part of that can also stay on the outside of the particle (Figure 5 (7)), depending on the thermal history of the individual particle. In this case, the carbon is present in both the resolidifying metal core and on the surface of the particle. Therefore, after cooldown and transition into *T*_S_, a crystalline structure is inhibited due to the incorporated carbon, stabilizing the amorphous structure of the HEA NP (Figure 5 (8)).

Looking at the diffusing carbon atoms during the period of *T*_L_, we additionally calculated the mean square displacements (MSD) of diffusing carbon atoms by using diffusion coefficients of carbon in FeNi at high temperatures and high pressures as reported by Lin et al. [99]. Using the estimated durations of 15 ns for ns-LAL and 2 ns for ps-LAL, the MSD falls within the one-digit nanometer range for both cases, though the value for ns-LAL is three times higher than that for ps-LAL. Notably, the diffusion coefficients reported by Lin et al. were studied under conditions that come closer to ns-LAL than to ps-LAL (regarding temperature and pressure). Since diffusion coefficients are inversely proportional to pressure and directly proportional to temperature, it is important to note that the pressure in ps-LAL can be ten times higher than that in ns-LAL while the temperature is only around twice as high. Consequently, we can expect that the MSD is even lower for ps-LAL, possibly falling in a sub-nanometer range, which makes diffusion into liquid metal droplets more unlikely. This hypothesized mechanism that takes carbon diffusion into the particle core into account extends the earlier stated assumption that amorphization is a result of a not-yet-crystallized structure which is stabilized by carbon shells throughout the cooling process [34]. Also, note that the aforementioned cooling rates of 10^12^–10^13^ K/s are crucial for preserving the amorphous structure as lower cooling rates would lead to the precipitation of carbon species to the surface of the particles, which was discussed by Abdelhafiz et al. in the context of high-entropy oxide materials via CTS [100]. Of course, carbon diffusion into the particle core after its cooldown may not be excluded and can even happen at room temperature. However, this is unlikely to be the driving factor as this may not explain the differences in the nanoparticle structure caused by ps- or ns-LAL which create particles of similar size. Also note that the estimated durations of *T*_L_ refer to simulations on silver; therefore, the durations for the Cantor alloy may differ due to lower thermal conductivity [101–102] and different electron–phonon-coupling strength.

**Figure 6:**
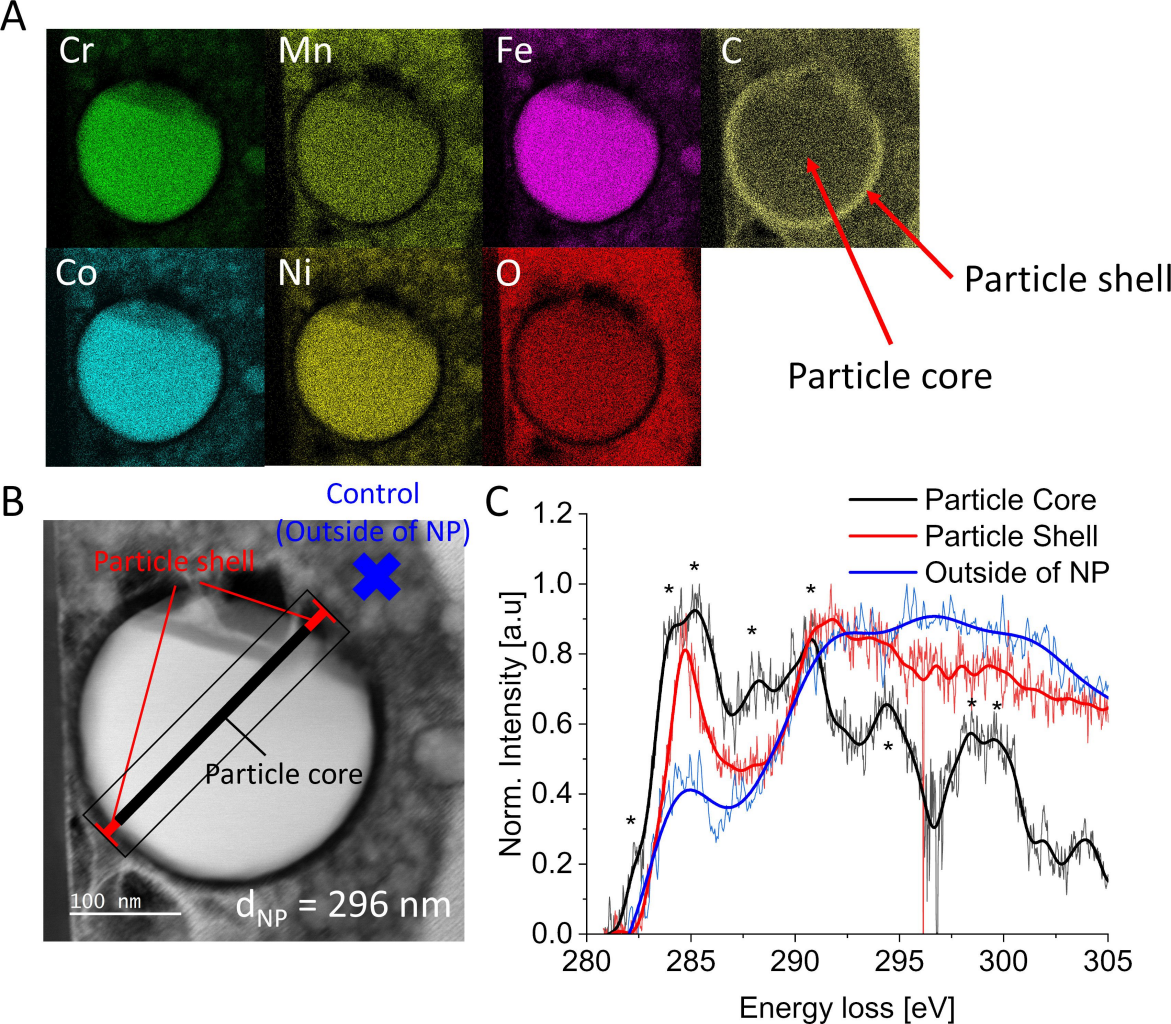
STEM-EDX analysis of a HEA NP cross section (synthesized via ns-LAL in acetone), prepared via FIB, highlighting evenly distributed metal atoms with additional oxygen and carbon signals inside the particle core with a carbon shell surrounding the HEA NP (A). EELS measurement of the FIB-prepared sample (B) with respective EELS spectra of particle shell and core (C) proves the presence of incorporated carbon inside the particle core. EELS signals of the particle shell resemble amorphous and graphitic carbon, while the signals in the particle core hint toward metal-carbon species [103–104]. The blue curve presents a measured control area outside of the HEA NP.

Although statistical quantification of carbon content inside the core structures of HEA nanoparticles requires further in-depth analysis with specialized analytical techniques, we can prove that carbon incorporation into HEA NPs via ns-LAL is observable as seen in Figure 6. We prepared a cross-section sample of an amorphous HEA NP from ns-LAL synthesis via focused ion beam (FIB) preparation and compared EELS signals originating from both the core and shell structure of the particle. Figure 6a highlights the elemental distribution of Cr, Mn, Fe, Co, Ni, O, and C throughout the cross section of the HEA NP. Here, evenly distributed metal atoms can be observed, surrounded by a carbon shell. However, note that carbon signals additionally originate from the particle core. The EELS measurement in Figure 6b and Figure 6c shows that both areas yield a significant amount of carbon signal. We observe that the particle shell mainly consists of amorphous and graphitic carbon (peak at 286 eV with respective σ* transition energy loss and possible peak at 294 eV), while the carbon species inside the particle core also resemble metal-carbon species [103–104], although a clear classification and quantification is difficult due to the presence of multiple elements. Thus, multiple interactions may lead to signal shifts that can easily be misinterpreted. Still, the combined techniques of sample preparation via FIB and consecutive EELS measurement strengthen our postulated carbon-diffusion-based mechanism about pulse-duration-driven stabilization of amorphous structures in ignoble HEA NPs.

## Conclusion

Understanding the structure formation determinants of high-entropy alloy nanoparticles is a prerequisite for further development of nanomaterial fabrication methods for applications in heterogeneous catalysis and magnetism. This suitability has been shown in earlier studies for high-entropy alloy nanoparticles, including laser-generated HEA NPs, in the application fields of catalysis and magnetism [1–2,34–35,105–107]. Note that the influence of graphitic shells on NPs, going along with LAL in organic solvents [54], has not been investigated yet in detail for laser-generated HEA NPs, although the results in the aforementioned studies show a high suitability for these applications despite the presence of carbon and oxidic surface species. Also, the presence of surface oxides, as found in our study, does not limit applicability, as surface oxides have been shown to positively affect [19] the catalytic process and oxy-hydroxides are frequently reported to be the catalytically active species [108] while in HEA NP electrocatalysis, such oxides are formed under anodic potential [19]. Also, the presence of surface manganese oxide still provides good catalytic activity with sharp performance profiles allowing for derivation of composition–activity correlations [18]. Furthermore, even though the size distributions reported in this work were not strictly monodisperse, size-separation techniques based on centrifugation are well established in the literature and could be applied to HEA NPs as well [109], even in a continuous operation mode using tubular bowl centrifuges [110]. Hence, despite minor inhomogeneities in mean particle size and composition on a single-particle level and the presence of thin graphite and oxide shells, the laser-fabricated ignoble HEA nanoparticles are promising candidates for applications in thermal and electro-catalysis and for potential applicability as magnets, where the near-limitless tunability of elemental composition could ease the development of new materials and help to elucidate structure–function correlations. Laser synthesis of colloids is a scalable nanofabrication method that provides kinetic control over the structural formation of nanoparticles due to the high cooling rates. On the other hand, less is known about the interplay with the liquid, in particular an organic liquid where the solvent molecule may deliver active carbon species which may form carbon shells or even act as a carbon dopant to nanoparticle cores of complex compositional alloys. We examined the primary factors influencing the formation of an amorphous phase in the quinary precious metal-free Cantor system (CrMnFeCoNi) synthesized by pulsed laser ablation in organic liquids. We found that the main determinant for the GFA during synthesis is the laser pulse duration, with ps pulses favoring crystalline structures and ns pulses triggering amorphous phase formation due to proposed carbon diffusion into the forming HEA NPs. Thereby, potential influencing factors such as particle size, target surface properties, nanoparticle composition, post-irradiation effects, and laser fluence were systematically excluded, leaving the determinant of pulse duration as the ruling factor in the structural difference after LAL in acetone, ethanol, and acetonitrile.

We further confirmed that the amorphous structure is metastable and the HEA NPs formed during LAL are formed under kinetic control. By conducting in situ heating and DSC experiments, we show that upon heating these particles to 375 °C (DSC) or 500 °C (TEM), activation energy barriers are overcome and phase transformation of the amorphous phase to the thermodynamically favored crystalline phase is observed. This outstanding temperature stability renders those nanosecond-laser-made particles to be interesting candidates for thermal catalysis or magnetic devices that pose high demand of structural robustness against heat.

This study advances the understanding of high-entropy alloy (or compositionally complex solid solutions) nanoparticles synthesized via laser ablation in organic liquids, highlighting how pulse duration sets structural differences. Technically, the switching between crystalline or amorphous nanoparticle outflow may easily be implementable, simply by switching a mirror that guides either a ps or ns pulsed laser beam into the ablation chamber. These findings are valuable for researchers in the field of laser-synthesized nanomaterials and the extension to other multi-element alloy systems would be of interest, enhancing knowledge of laser-based synthesis of (high-entropy alloy) nanoparticles. In this context, it would be interesting to examine further whether or to what extent amorphization tendencies are linked to the carbon affinity of individual elements in the alloy. Additionally, the application potential of amorphous nanoparticles is promising, such as high intrinsic activity in both electrocatalysis and thermal catalysis, where their disordered atomic structure provides a high density of active sites, which enhances their catalytic performance. Furthermore, in the field of energy storage devices, such as batteries and supercapacitors, such amorphous nanoparticles are interesting materials. Here, the combination of complex composition based on the HEA concept with carbon doping elevating the glass-forming ability of the Cantor composition could be an interesting playground, in particular as i) the formed particles have outstanding temperature stability, and ii) the LAL is a fabrication method with well-documented scalability and robustness.

## Experimental

### HEA nanoparticle synthesis

For HEA NP synthesis, the corresponding ablation target with a nominal composition of Cr_20_Mn_20_Fe_20_Co_20_Ni_20_ was produced by weighing and heat-treating metal granules of Cr, Mn, Fe, Co Ni (Evochem, purity 99.95–99.99%) inside an arc-melting oven in an argon atmosphere for melting and sintering. The inert atmosphere was used to avoid oxidation of the ignoble metals used. The sintered target was remelted three times to ensure homogeneity and uniform phase formation.

The surfactant-free HEA NPs were LAL-synthesized in acetone, ethanol, and acetonitrile (VWR, purity ≥99.8%). Before ablation, the bulk target was polished with sandpaper to ensure it was free of surface oxides. All commercially available analytical grade solvents (purity ≥99.8%) were purified by dewatering (molecular sieve 4 Å, Carl Roth), distillation, and subsequent degassing with argon to minimize contamination and oxidation of the HEA NPs. The ablation was conducted in a self-designed stirred batch reactor with a volume of 30 mL using an Nd:YAG laser (Ekspla, Atlantic Series, 10 ps, 1064 nm, 100 kHz, 0.15 mJ) for ps-LAL and a nanosecond Laser (Rofin, Powerline E20, 10 ns, 1064 nm, 10 kHz, 0.50 mJ) for ns-LAL. Ablation was conducted for 5 min. The laser beam was moved on the target with a galvanometric scanner (100 mm focal length) in a spiral pattern, while lateral inter-pulse distances were set to avoid interactions between the cavitation bubbles and consecutive pulses.

**Table 1:** Overview of the pulsed lasers used in this work with all relevant system parameters.

Laser	Picosecond	Nanosecond

pulse duration	10 ps	10 ns
wavelength	1064 nm	1064 nm
repetition rate	100 kHz	10 kHz
pulse energy	0.15 mJ	0.50 mJ

For evaluation and application of laser fluences, a Baumer USB camera was used with the help of the Laser Light Inspector software, applying a Gaussian fit and using the 1/e^2^ method to determine laser beam diameters. For laser power, a power meter (PowerMax PM30, Coherent) was used, measuring the average laser power behind all optics.

Post-irradiation control experiments were conducted in standard quartz glass cuvettes (Hellma Analytics, High Precision Cell) with a colloid volume of 3 mL. Focusing conditions and irradiated volumes in the cuvette closely mimicking those in the ablation chamber were realized (Supporting Information File 1, Figure S1). The colloids, whether made by ps-LAL or ns-LAL, were irradiated in a colloidal state by the respective lasers. This allowed us to emulate and investigate the contribution of the by-process of post-irradiation of colloids in the liquid volume in front of the ablation target during LAL.

### Material characterization methods

Target characterization was conducted by X-ray powder diffraction (Bruker D8 Advance, Cu Kα with λ = 1.54 Å) in reflection mode in a 2θ range 5 to 130° with a step size of 0.01° and a counting time of 1.2 s, scanning electron microscopy with energy dispersive spectroscopy (Philips XL30 with EDAX system) analysis as well as X-ray fluorescence spectroscopy (S8 Tiger, Bruker) to confirm global composition, elemental distribution and crystal structure. HEA NPs were characterized via XRD by drying drop cast, whereby concentrated colloidal HEA NPs with comparable masses were placed on a Si single-crystal sample holder to minimize scattering. The measurements were performed with the same diffractometer (Bruker D8 Advance) in a 2θ range of 20° to 90° with a step size of 0.02° and a counting time of 8 s. For the qualitative phase analysis, the Bruker software Diffrac Suite EVA V7.1 was used with the face-centered cubic Ni (#70-1849) and MnO (#75-0257) pattern from the ICDD database. To calculate the lattice parameters and the average crystallite size, a quantitative Rietveld refinement was performed with the Bruker software TOPAS 7.0, after the instrumental characterization with a microcrystalline powder LaB_6_ (SRM 660b of NIST, *a* = 4.15689 Å) was done.

TEM analysis including SAED (*d*-value determination errors within ±0.01 Å range) and HRTEM was performed using a Tecnai F30 STwin G2 (300 kV acceleration voltage) equipped with a Si(Li) detector (EDAX system) and a JEOL JEM 2100 (200 kV acceleration voltage). Chemical analysis involving elemental mapping and line scanning was conducted at a probed-corrected JEOL JEM-ARM200F NEOARM scanning transmission electron microscope operated at 200 kV (cold-FEG) using EDX with a dual silicon drift detector system with 100 mm^2^ active area each. Additionally, in situ heating experiments were performed utilizing TEM and SAED also at the same JEOL NEOARM provided with a Lightning HB+JEOL holder from DENS solutions. All samples for TEM analysis were prepared by drop casting the NP colloid on copper grids with a lacey carbon film (Plano GmbH) or silicon nitride films (TED Pella Inc. 35 nm, 70 × 70 µm aperture). For in situ heating experiments, a wildfire Nano-Chip GT from DENS solutions was used as a substrate. After drop casting, all samples were dried in the atmosphere using an infrared lamp (Philips Infrared PAP38E, 150 W) for 1 min and permanently stored under vacuum to avoid further contamination and oxidation. For particle size histograms, TEM overview images were analyzed with the ImageJ software to measure particle diameters. All histograms were fitted using a LogNormal distribution. The cross-section sample was prepared by drying 40 µL of the particle solution for 3 h at 200 °C on a Si-wafer piece. Before cutting, a Pt protective layer was sputtered on top. Afterwards, it was cut by FIB using a standard lift-out method with a FEI Helios Nanolab system.

Differential scanning calorimetry was conducted on the 204 Cell (Netzsch) with a heating rate of 1 K/min and MnCl_3_ as a reference material to determine phase transitions in amorphous high-entropy alloy nanoparticles. To avoid unwanted processes, an inert atmosphere was created by flushing the measurement chamber with argon gas.

## Supporting Information

File 1 Additional figures and tables.

File 2 This video highlights thermal induced phase transformation of amorphous HEA NPs via ns-LAL into the thermodynamically favored crystalline fcc structure.

## Data Availability

Data generated and analyzed during this study is available from the corresponding author upon reasonable request.
