# Supplementary material for: Crystalline and amorphous structure selectivity of ignoble high-entropy alloy nanoparticles during laser ablation in organic liquids is set by pulse duration
Source: Beilstein J Nanotechnol. 2025 Jul 17;16:1141–59. doi: 10.3762/bjnano.16.84 (PMC12278108; doi:10.3762/bjnano.16.84)
Supplement: File 1 — Additional figures and tables. [file Beilstein_J_Nanotechnol-16-1141-s001.pdf]

BEILSTEIN JOURNAL OF NANOTECHNOLOGY

Supporting Information

for

Crystalline and amorphous structure selectivity of ignoble high-entropy alloy nanoparticles during laser ablation in organic liquids is set by pulse duration

Robert Stuckert, Felix Pohl, Oleg Prymak, Ulrich Schürmann, Christoph Rehbock, Lorenz Kienle and Stephan Barcikowski

Beilstein J. Nanotechnol. **2025,** *16,* 1141–1159. doi:10.3762/bjnano.16.84

Additional figures and tables

License and Terms: This is a supporting information file under the terms of the Creative Commons Attribution License (https://creativecommons.org/licenses/by/4.0). Please note that the reuse, redistribution and reproduction in particular requires that the author(s) and source are credited and that individual graphics may be subject to special legal provisions.
The license is subject to the *Beilstein Journal of Nanotechnology* terms and conditions: (https://www.beilstein-journals.org/bjnano/terms)

S1. Schematic illustration of the batch ablation chamber

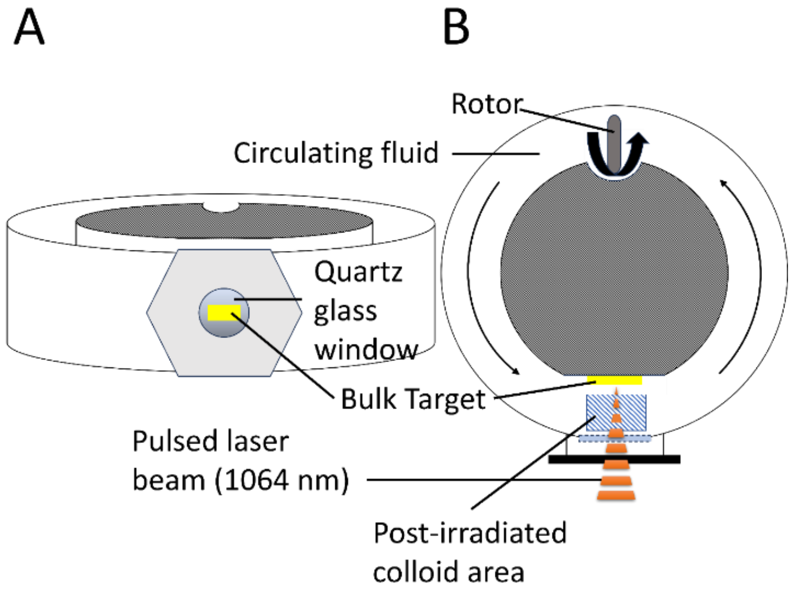

Figure S1: Schematic representation of the ablation chamber used in this study, shown from the front (A) and from the top (B). The circulating fluid represents the respective solvents acetone, ethanol, and acetonitrile.

S2. Global compositional values determined via STEM-EDX and respective deviations

Table S1: EDX measurements of crystalline CrMnFeCoNi nanoparticles (via picosecond-pulsed LAL) at five different positions with average values and standard deviations (St.Dev.).

Position	Cr-K [at%]	Mn-K [at%]	Fe-K [at%]	Co-K [at%]	Ni-K [at%]
**1**	22.8	17.7	21.2	18.1	20.3
**2**	22.3	16.9	21.8	18.7	20.3
**3**	22.6	17.0	20.8	20.0	19.6
**4**	23.5	14.0	21.1	20.4	21.0
**5**	19.7	18.3	21.0	22.5	18.6
**Average**	22.2	16.8	21.2	19.9	20
**St.Dev.**	1.31	1.49	0.36	1.52	0.83

Table S2: EDX measurements of amorphous CrMnFeCoNi nanoparticles (via nanosecond-pulsed LAL) at five different positions with average values and standard deviations (St.Dev.).

**Position**	**Cr-K [at%]**	**Mn-K [at%]**	**Fe-K [at%]**	**Co-K [at%]**	**Ni-K [at%]**
**1**	23.5	20.3	18.9	18.2	19.1
**2**	20.8	20.9	20.1	20.4	17.7
**3**	21.5	21.5	18.5	18.9	19.6
**4**	18.2	22.4	17.8	20.1	21.5
**5**	20.7	20.6	21.8	18.1	18.8
**Average**	20.9	21.1	19.4	19.1	19.3
**St.Dev.**	1.70	0.74	1.40	0.95	1.25

S3. Characterization of the CrMnFeCoNi bulk target

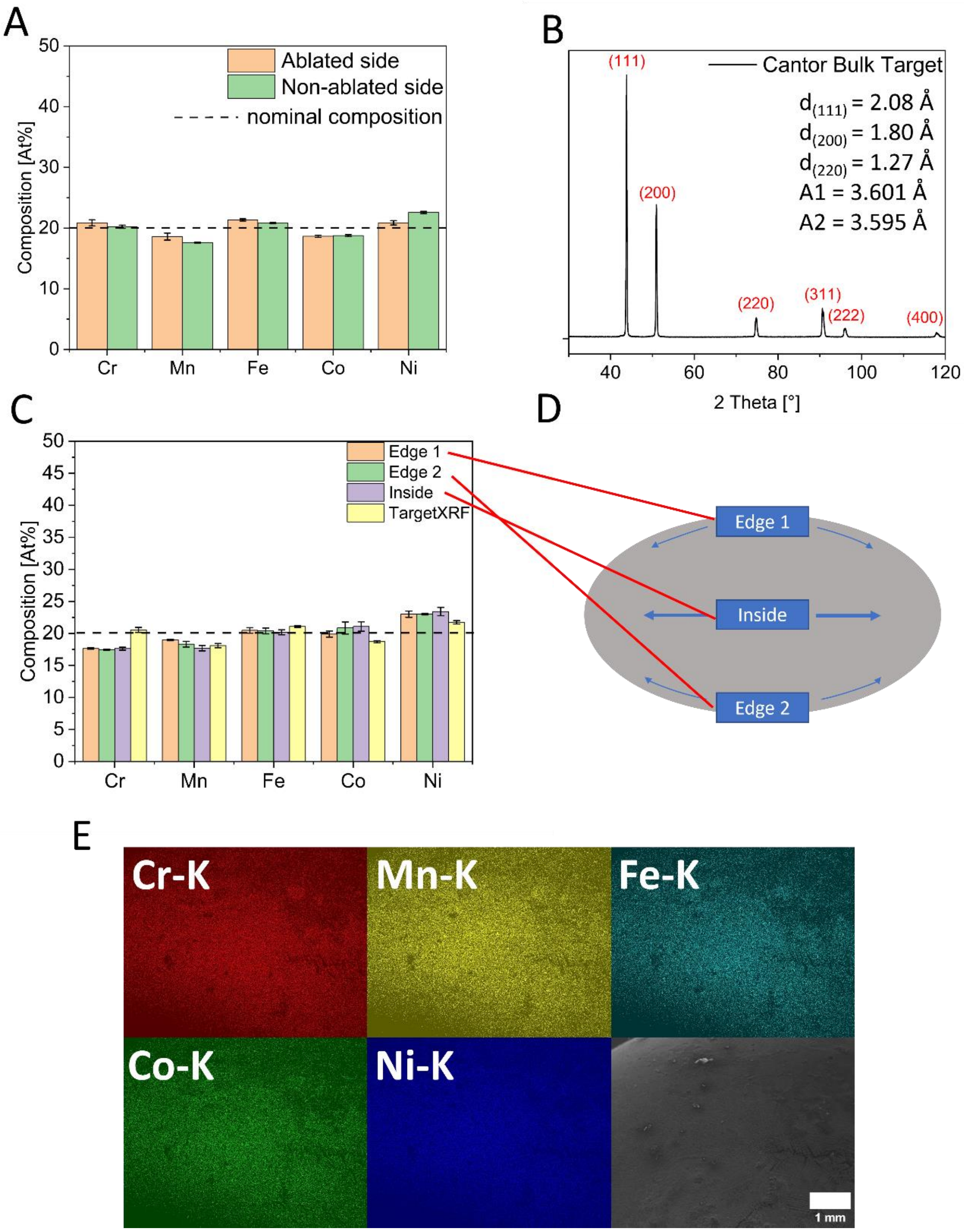

Figure S2: (A) XRF-analysis of both of the bulk target surfaces compared to the nominal composition. (B) XRD pattern of the bulk target with reflections at 2θ = 43.8°, 51.0°, 74.8°, 90.6°, 95.4°, and 117.8, and respective *d*-values as well as the lattice parameters of 3.601 Å and 3.595 Å, depicting an fcc structure. (C) EDX data of the bulk target cross-section (shown in (D)). Both edges and inside values are determined by five EDX measurements each and are compared to the global XRF values, shown

in (A). (E) EDX map of a selected area showing the homogeneous distribution of the elements Cr, Mn, Fe, Co, and Ni. Dark spots are attributed to surface contaminations and can be removed by sandpaper. Error bars in (A) and (C) represent standard deviation of three measurements in the XRF analysis and five measurements in teh EDX analysis.

S4. CrMnFeCoNi colloids made in acetonitrile (ps-LAL) and ethanol (ns-LAL)

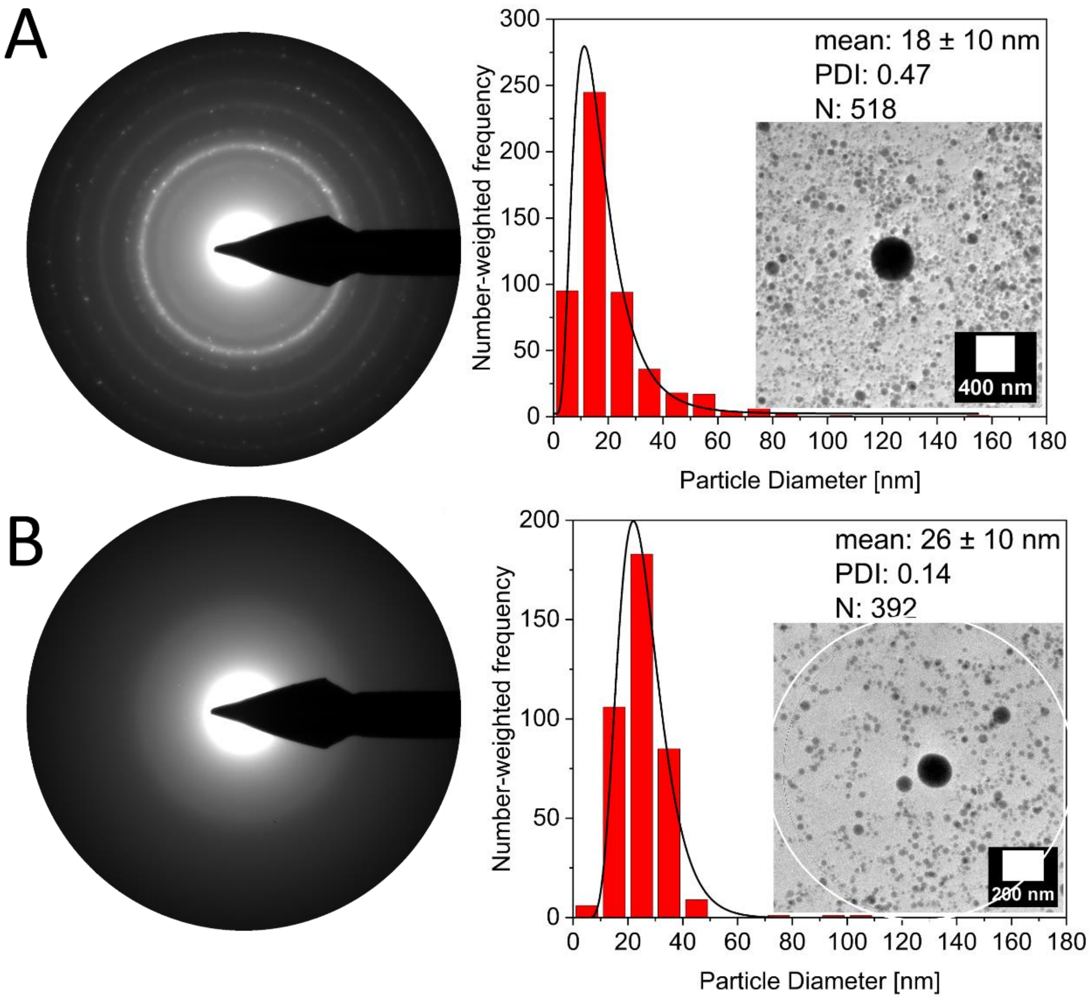

Figure S3: SAED analysis of crystalline colloids made by ps-LAL in acetonitrile (A) and amorphous colloids by ns-LAL in ethanol (B) to extend the studies by Waag et al. [1] and Johny et al. [2]. The corresponding areas used for SAED analysis and particle size distribution are marked with a white circle inside the overview TEM images. N = number of particles, PDI = polydispersity index.

S5. HAADF image and EDX analysis of HEA NPs with an oxide shell

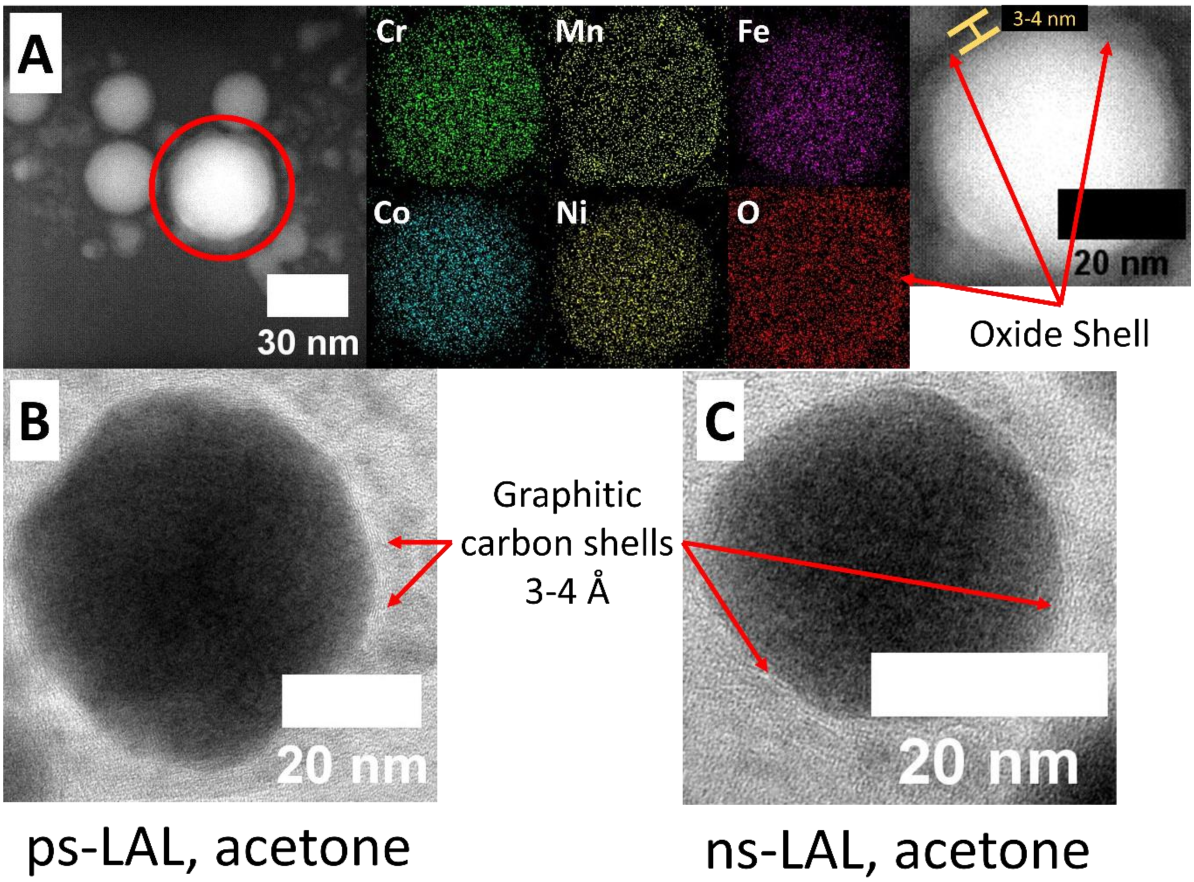

Figure S4: HAADF image and EDX analysis of a HEA NP with an oxide shell that correlates with the Mn signal of the EDX analysis, being one possible explanation for the appearance of MnO signals in the XRD spectra. (A) HRTEM images of chosen HEA NPs with graphitic shells, synthesized by ps-LAL in acetone (B) and ns-LAL in acetone (C).

S6. Crystalline vs amorphous structure in CrMnFeCoNi colloids made by ps-LAL/ns-LAL in ethanol

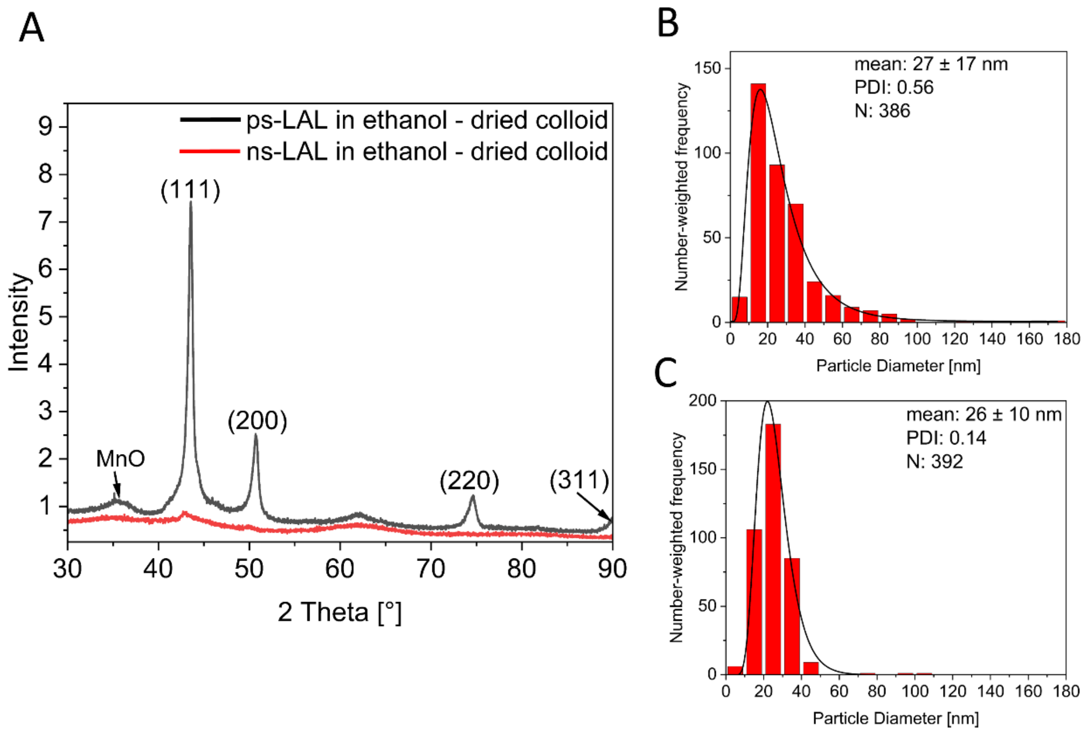

Figure S5: XRD pattern, comparing CrMnFeCoNi colloids made by both ps- and ns-LAL in ethanol, strengthening the observation of solvent-independent structural differences in ignoble HEA NPs. The crystalline particles by ps-LAL depict two fractions oflattice parameters (3.599 Å and 3.615 Å) with crystallite sizes of 25 nm and 9 nm, respectively (A). Representative particle size distributions of HEA NPs by ps-LAL in ethanol (B) and by ns-LAL (C), showing the same trend in polydispersity as in acetone.

S7. SEM-EDX analysis of post-irradiated bulk target surface after ps- and ns-LAL

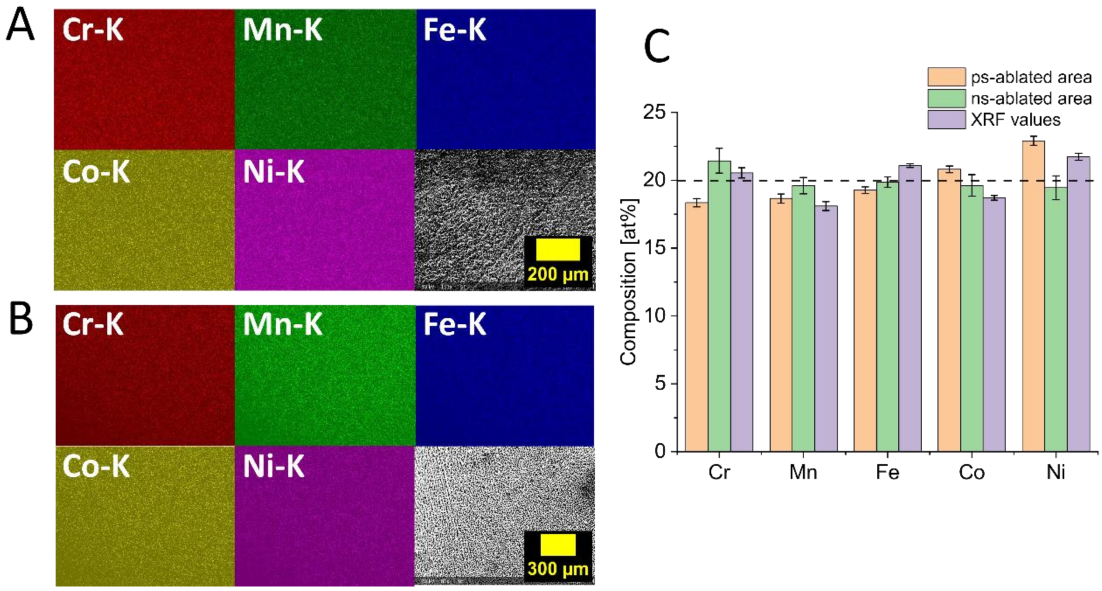

Figure S6: EDX analysis of post-ablated target surfaces after ps-LAL (A) and ns-LAL (B) in acetone. (C) Average composition of five measurements each, comparing values of both ps- and ns-ablated surface with global XRF values, averaged from Figure S2 A.

S8. Compositions of single HEA NPs

Section S10 provides data about the composition of representative HEA NPs obtained by LAL in acetone. In both tables particles were chosen which are comparable in size to the determined mean values of 21 ± 10 nm for ps-LAL and 21 ± 7 nm for ns-LAL, particles that exceed the mean particle diameter by roughly the double value, and particles that exceed the mean particle diameter by at least four times. In both cases a lack of manganese is obvious, even though in ns-LAL the depletion is not as strong as in ps-LAL.

Table S3: Composition of individual HEA NPs made by ps-LAL in acetone.

Particle	Diameter	Cr [at%]	Mn [at%]	Fe [at%]	Co [at%]	Ni [at%]
**1**	18	29	8	22	23	18
**2**	22	19	15	20	20	26
**3**	23	21	8	23	23	25
**4**	31	19	11	22	24	24
**5**	36	22	10	22	23	23
**6**	39	28	13	21	20	18
**7**	40	22	12	23	21	22
**8**	40	21	14	22	23	19
**9**	42	21	12	23	23	21
**10**	46	18	17	23	21	18
**11**	49	21	14	20	21	24
**12**	57	20	16	22	22	20
**13**	80	20	16	20	21	24
**14**	117	19	15	23	22	20
**Average**		**21**	**13**	**21**	**22**	**23**
**St.Dev.**	**/**	**3.1**	**2.8**	**1.1**	**1.2**	**2.7**

Table S4: Composition of individual HEA NPs made by ns-LAL in acetone.

Particle	Diameter [nm]	Cr [at%]	Mn [at%]	Fe [at%]	Co [at%]	Ni [at%]
**1**	18.0	19	16	19	22	24
**2**	21.0	19	27	17	19	18
**3**	25.0	24	15	22	21	18
**4**	26.0	19	23	20	18	20
**5**	27.0	21	16	20	21	22
**6**	28.0	26	14	23	21	16
**7**	29.0	25	15	22	20	16
**8**	33.0	23	17	21	20	19
**9**	35.0	23	16	22	20	19
**10**	37.0	24	14	22	21	19
**11**	42.0	23	20	22	18	17
**12**	49.0	23	26	22	21	18
**13**	126.0	22	10	24	23	21
**14**	159.0	27	11	24	21	17
**Average**		**22**	**17**	**21**	**21**	**19**
**St.Dev.**		**2.4**	**4.9**	**1.8**	**1.3**	**2.2**

S9. Parameters and crystallographic results conducted by experiments at the same laser fluence

Table S5: Laser parameters used for conducting control experiments in LAL with the same laser fluences of 1.6 J/cm² (control experiment 1) and pulse energy of 0.15 mJ (control experiment 2). Ps-LAL at a pulse energy of 0.15 mJ is shown in the main manuscript.

**Control Experiment 1**		
	**ps-LAL**	**ns-LAL**
**Wavelength**	1064 nm	1064 nm
**Pulse Duration**	10 picoseconds	10 nanoseconds
**Frequency**	100 kHz	10 kHz
**Mean Power**	4.2 W (0.04 mJ)	1.2 W (0.12 mJ)
**Area of focused laser beam**	$2.6 \cdot 10^{-3}$ mm²	$7.2 \cdot 10^{-3}$ mm²
**Laser Fluence**	**1.6 J/cm²**	**1.6 J/cm²**

**Control Experiment 2**	
**Wavelength**	1064 nm
**Pulse Duration**	10 nanoseconds
**Mean Power**	5.0 W
**Frequency**	33.3 kHz
**Pulse Energy**	**0.15 mJ**

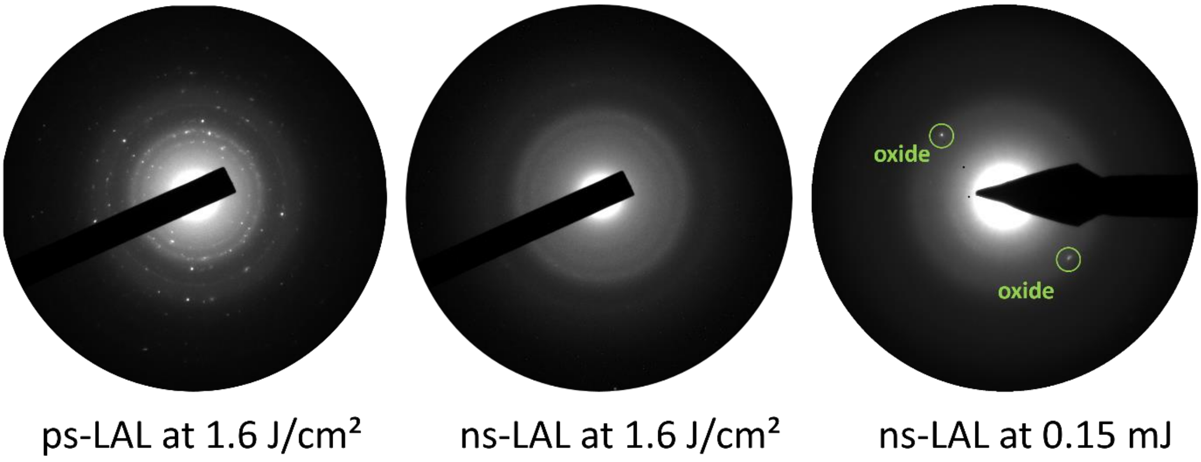

Figure S7: SAED analysis of ensemble HEA NPs made by LAL in acetone. Control experiments 1 show ps-LAL at 1.6 J/cm² (left) and by ns-LAL at 1.6 J/cm² (center). Control experiment 2 (right) shows SAED analysis of ensemble HEA NPs via ns-LAL at the same pulse energy (0.15 mJ) used during initial ps-LAL syntheses in this study. Marked reflections result from (FeMn)O oxides (d = 2.18 Å ICSD: 60687): These experiments confirm that the pulse-duration-driven structural heterogeneity is not a result of varying fluences/pulse energies but analogously is observable at adapted parameters.

S10. SAED analyses after post-irradiation experiments of already produced colloids

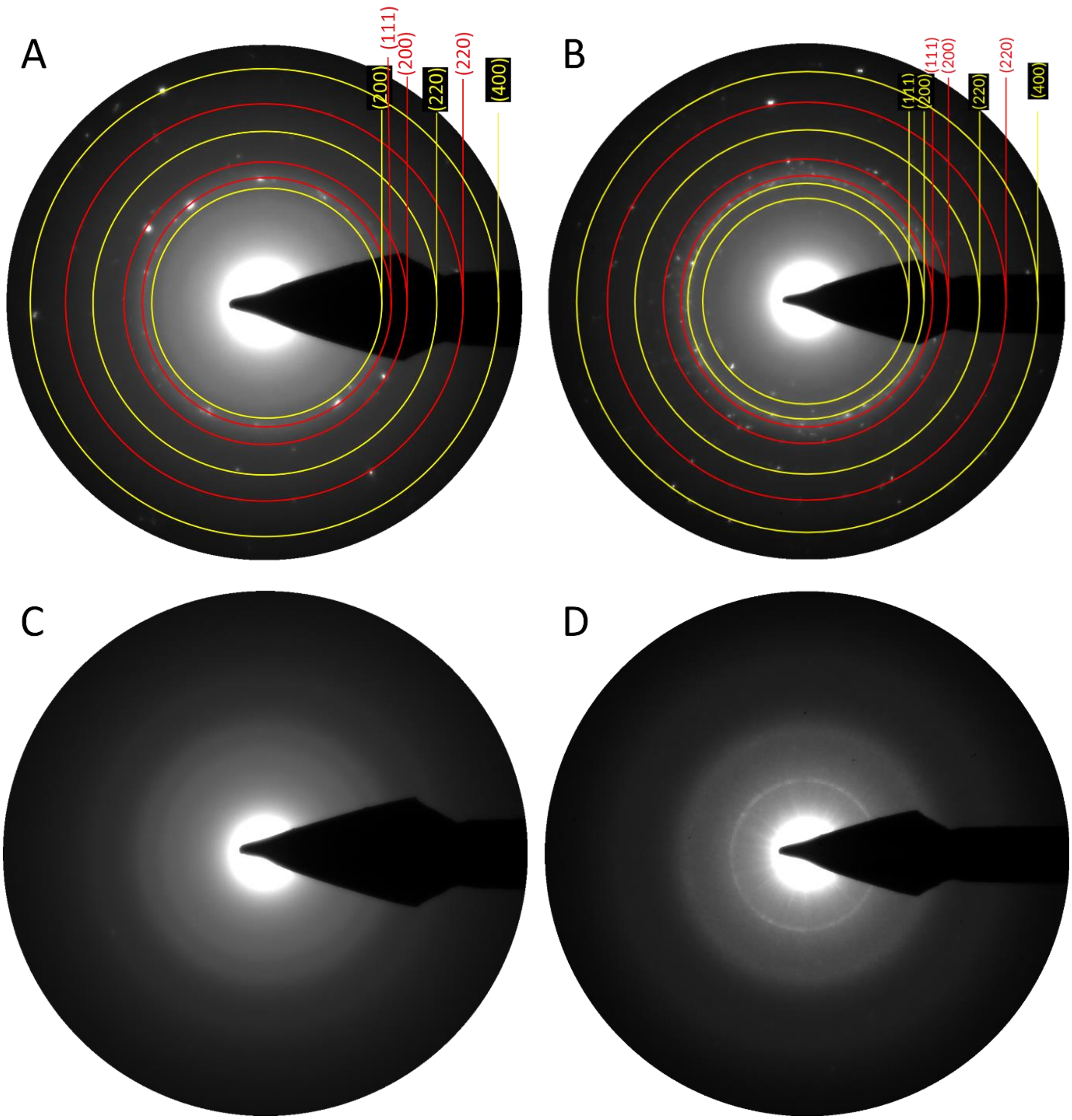

Figure S8: SAED analysis of post-irradiated colloids in acetone. (A) Colloids by ps-LAL irradiated with ns pulses. (B) Colloids by ps-LAL irradiated by ps pulses. (C) Colloids by ns-LAL irradiated by ns pulses. (D) Colloids by ns-LAL irradiated by ps-pulses. Red circles represent the respective reflections from the metallic fcc structure while yellow circles represent manganese-dominated oxide species. The results show that post-irradiation exhibits no effect on the structure of the nanoparticles.

S11 Long-term stability and batch-to-batch reproducibility of HEA NPs

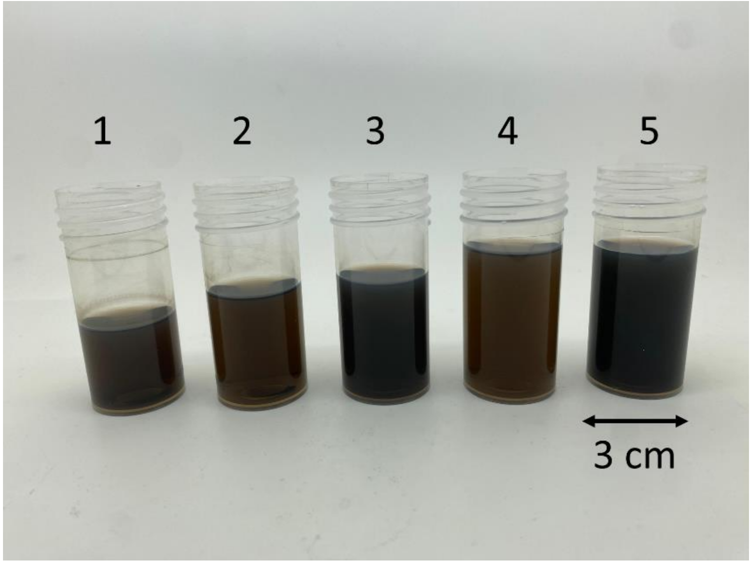

Figure S9: Image of colloids from this work, stored for at least three months after synthesis, highlighting colloidal stability of laser-generated high-entropy alloy nanoparticles in organic solvents. Displayed are Cantor alloys: (1) in acetone after ps-LAL, (2) in acetone after ns-LAL, (3) in ethanol after ps-LAL, (4) in ethanol after ns-LAL, and (5) in acetonitrile after ps-LAL.

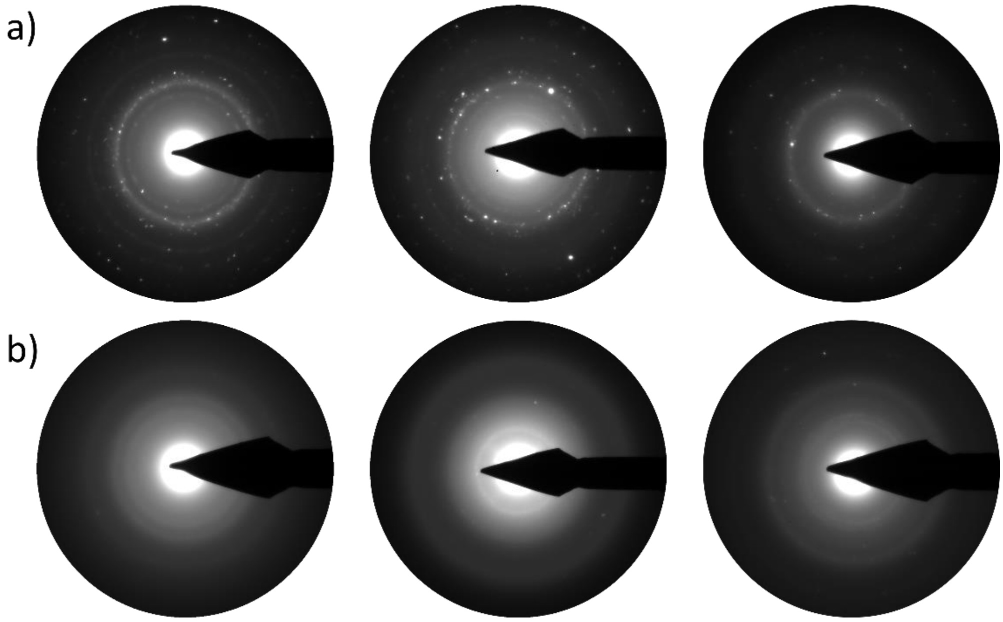

Figure S10: Structural reproducibility of laser-generated Cantor HEA NPs in acetone made via ps-LAL (a) and via ns-LAL (b), showing SAED images of three different samples in each row. This confirms that the structural difference after ps-LAL and ns-LAL is a reproducible observation.

References

1. Waag, F.; Li, Y.; Ziefuß, A. R.; Bertin, E.; Kamp, M.; Duppel, V.; Marzun, G.; Kienle, L.; Barcikowski, S.; Gökce, B. *RSC Adv.* **2019,** *9*, 18547–18558. doi:10.1039/C9RA03254A
2. Johny, J.; Li, Y.; Kamp, M.; Prymak, O.; Liang, S.-X.; Krekeler, T.; Ritter, M.; Kienle, L.; Rehbock, C.; Barcikowski, S.; Reichenberger, S. *Nano Res.* **2022,** *15*, 4807–4819. doi:10.1007/s12274-021-3804-2
